# Peppermint oil exhibits antispasmodic and anti-inflammatory properties and alters gut microbial community composition and gas production *in vitro*

**DOI:** 10.3389/fphar.2026.1963380

**Published:** 2026-10-22

**Authors:** Christian Buettner, Philipp Peterburs, Anja Schulz-Kuhnt, Lars Herrmann, Michael Soeberdt, Christoph Abels

**Affiliations:** Bionorica SE, Neumarkt in der Oberpfalz, Germany

**Keywords:** anti-inflammatory, antispasmodic, BNO 2910, Digestopret^®^, gut microbiome, irritable bowel syndrome, menthol, peppermint oil

## Abstract

**Background:**

Peppermint oil (PMO) is an herbal medicinal product used for the symptomatic relief of minor spasms of the gastrointestinal tract, flatulence and abdominal pain, especially in patients with irritable bowel syndrome (IBS). To gain a more comprehensive understanding of the multimodal effects of PMO in IBS, the investigations aimed to confirm its antispasmodic and anti-inflammatory effects and assess direct effects of PMO on the human gut microbiome and microbial gas production *in vitro*.

**Methods:**

To investigate the spasmolytic activity of PMO (BNO 2910), circular smooth muscle strips from the proximal colon of five male guinea pigs were used. Its anti-inflammatory potential was evaluated using RAW 264.7 macrophages stimulated with LPS. TNF secretion and nitrite levels, as an indicator of NO production, were measured using ELISA and Griess reagent, respectively. Effects on the human gut microbiome and colonic fermentation activity were assessed in an anaerobic *in vitro* fermentation system inoculated with stool samples from six healthy donors using 16S rRNA gene sequencing, flow cytometry-based cell counting and gas production measurements.

**Results:**

PMO showed antispasmodic effects on isolated smooth muscle strips of guinea pig colon tissue as well as inhibition of LPS-induced TNF secretion and NO induction in RAW 264.7 macrophages. Additionally, PMO significantly reduced microbial gas production in stool samples from healthy donors, indicating reduced microbial fermentation activity under the experimental conditions, while the alpha diversity indices richness and evenness were maintained or even increased. At the genus level, PMO showed direct effects on microbiome community composition, inhibiting the growth of potential disease-related organisms (e.g., *Escherichia-Shigella* or *Eggerthella)*, while maintaining or even increasing the abundance of health-related organisms (e.g., *Blautia* and *Faecalibacterium*).

**Conclusion:**

Besides antispasmodic and anti-inflammatory effects, PMO exhibits direct effects on the human gut microbiome, including the modulation of microbial communities and total gas production *in vitro*. To determine the specific relevance of the observed microbiome-related effects of PMO in IBS further disease-specific studies are warranted.

## 1 Introduction

Irritable bowel syndrome (IBS) is a common disorder of gut-brain interaction. Prevalence varies depending on country and applied diagnostic criteria but is estimated at approximately 10% globally, with a higher prevalence in developed countries (Canavan et al., 2014; Oka et al., 2020; Arif et al., 2025). Currently, no IBS-specific diagnostic test is available (Black and Ford, 2025). Instead, diagnosis is symptom-based according to the recently updated Rome V criteria, which define IBS as a disorder of gut-brain interaction characterized by recurrent but not continuous abdominal pain or discomfort in association with disturbed bowel habits (e.g., constipation, diarrhea, or a combination of both) for several months (Lacy and Patel, 2017; Layer et al., 2021; Drossman et al., 2026). Further typical symptoms include gastrointestinal spasms, bloating, and flatulence (Enck et al., 2016; Lacy and Patel, 2017; Black and Ford, 2020). Abdominal pain is often considered one of the most burdensome IBS symptoms and can be caused by abdominal spasms or bloating due to excessive gas in the digestive tract (Cain et al., 2006; Layer et al., 2021).

To date, the complex pathomechanism of IBS has not been fully understood. Studies suggest that IBS is the result of a multifactorial interplay of various dysfunctions. These alterations cause impaired epithelial integrity and function, gut microbial dysbiosis, visceral hypersensitivity, altered gut motility, low-grade intestinal inflammation, and an altered gut-brain axis (Ford et al., 2020; Sun et al., 2025).

Due to the multifactorial pathogenesis of the disease, effective treatment demands the targeting of several dysfunctions at once. Current therapies are symptom-based and include non-pharmacological approaches, such as lifestyle modifications and psychotherapy. First-line pharmacological therapies include laxatives, antidiarrheals, and antispasmodics, such as peppermint oil (PMO; *Mentha x piperita* L., aetheroleum), which is recommended by several guidelines (Pietrzak et al., 2018; Kindt et al., 2022). Probiotics are considered another treatment option in IBS to directly target the dysbiosis of the gut microbiome (Layer et al., 2021).

Efficacy and safety of PMO for the treatment of IBS is clinically established (Capanni et al., 2005; Cappello et al., 2007; Ford et al., 2008; Alammar et al., 2019; Ingrosso et al., 2022) with PMO being especially effective in relieving global IBS symptoms and abdominal pain (Committee on Herbal Medicinal Products, 2020a; Ingrosso et al., 2022). Furthermore, a significant improvement in bloating upon treatment with PMO was suggested (Cappello et al., 2007; Cash et al., 2016; Mosaffa-Jahromi et al., 2016). Even though some recent clinical trials with PMO in adults and children with functional gastrointestinal disorders reported negative primary endpoints (Weerts et al., 2020; Vermeijden et al., 2026), recent meta-analyses support PMO as effective treatment in IBS (Ingrosso et al., 2022; Meyer et al., 2026).

PMO and its main active component, L-menthol, exert several pharmacodynamic effects that may explain the clinical efficacy of PMO in IBS. The best described and assumed principal mode of action is a dose-dependent antispasmodic effect on intestinal smooth muscles (Chumpitazi et al., 2018), mainly mediated via a reduction in Ca^2+^ influx. This effect has been demonstrated for both PMO (Hills and Aaronson, 1991; Committee on Herbal Medicinal Products, 2020a; Krueger et al., 2020) and its main component L-menthol (Hawthorn et al., 1988; Committee on Herbal Medicinal Products, 2020a). Additional proposed mechanisms include choleretic, anti-foaming and analgesic properties as well as antioxidant and anti-inflammatory activities (Chumpitazi et al., 2018; Committee on Herbal Medicinal Products, 2020a). Furthermore, PMO may influence the dysbiotic gut microbiome (Moniruzzaman et al., 2025), which is particularly relevant when PMO is administered as enteric-coated preparation, enabling direct interaction with the microbial community. However, the potential interactions between PMO and the human gut microbiome are yet to be adequately investigated. To gain a more comprehensive image on the multimodal effects of PMO in IBS, the present study focused on three central mechanisms implicated in IBS pathophysiology, namely, intestinal spasms, low-grade inflammation and gut microbiome alterations. As anti-inflammatory and antispasmodic activities represent established pharmacological properties of PMO, these experiments were included as supportive models. In contrast, the primary objective of the present study was to investigate direct effects of PMO on the human gut microbiome.

## 2 Materials and methods

### 2.1 Test item

PMO (BNO 2910, *Mentha x piperita* L., aetheroleum) was obtained from Düllberg Konzentra GmbH & Co. KG, Hamburg (Germany) (Batch-No.: 0000189256) and its chemical composition is in line with the European Pharmacopoeia monograph (Council of Europe, 2023). The tested PMO contained the following main components: 43.2% menthol, 25.5% menthone, 0.0% carvone, 5.8% 1.8-cineole, 3.9% isomenthone, 0.1% isopulegole, 2.4% limonene, 1.5% menthofuran, 5.4% menthyl acetate, and 0.9% pulegone. Moreover, the tested PMO is in line with the HMPC monograph and assessment report on *Mentha x piperita* L., folium and aetheroleum (Committee on Herbal Medicinal Products, 2020a; Committee on Herbal Medicinal Products, 2020b).

### 2.2 Spasmolytic activity of PMO in guinea pig colon tissue

Male Dunkin-Hartley guinea pigs (300–350 g) were purchased from Charles River Laboratories (France). All experiments using guinea pig colon tissue were performed in accordance with French legislation and in accordance with a valid license for experiments on vertebrate animals, issued by the French Ministry for Agriculture and Fisheries. The study was conducted in accordance with local legislation and institutional requirements. Experiments were performed according to the protocols of Urosphere (France). In brief, guinea pigs were euthanatized by CO_2_ asphyxiation. The proximal colon was excised and cleaned. For preparation of circular smooth muscle strips (10 mm × 5 mm), the mucosa was carefully removed by gently scraping the tissue with a coverslip while maintaining the integrity of the myenteric plexus and longitudinal muscle layer. For each test condition five muscle strips were prepared from different guinea pigs. The resulting muscle strips were then tied with cotton threads to secure them between the holder and the isometric transducer (it-1, EMKA Technologies, France) and mounted, under 10 mN (1 g) initial tension in 5 mL organ baths (EMKA Technologies, France) containing oxygenated Krebs-Henseleit solution, pH = 7.4 and kept at 37 °C. Contractile responses were recorded using Chart® software version 7.3.7 and PowerLab 8/35 data acquisition system (ADInstruments Pty Ltd., Australia). After at least 60 min of equilibration, during which the Krebs solution was replaced every 15 min and the tension was readjusted, PMO (final concentrations of 10, 30 and 100 μg/mL), its common solvent (a corresponding volume of dimethyl sulfoxide, DMSO, 0.1%, arecoline (contractile control; 0.01, 0.1 and 1 μM; batch n° A0407407, Acros Organics (Belgium)) or forskolin (relaxant control; 0.01, 0.1 and 1 μM; batch n° 4A, Tocris (United Kingdom)) (all at non-cumulative concentrations) was added to each strip. After obtaining a maximal effect but at least after 15 min of recording, the tissues were washed every 10 min during an interval period of 30 min, then pre-contracted again at 10 mN of tension before testing the next concentration. To ensure tissue viability, a reference contraction induced by 80 mM KCl (H_2_O) was performed. At the end of the experiment, a wash-out of 30 min was applied to verify if the effects of the test substance can be reversed. Finally, a reference contraction to 80 mM KCl was performed on each strip to evaluate tissue viability.

### 2.3 RAW 264.7 macrophage culture and determination of TNF secretion and NO induction

RAW 264.7 macrophages (14th, 18th and 12th passages for three independent experiments) were provided by the University of Cordoba, Spain. Cells were maintained in supplemented DMEM medium (Gibco, United States) containing 10% FBS (Anprotec, Germany) and 1% penicillin/streptomycin (Gibco, United States) at 37 °C in a humidified atmosphere of 5% CO_2_. A stock solution of PMO was prepared in 100% DMSO. Further dilutions were made in cell culture medium (DMEM). Before measuring TNF secretion or NO induction, non-cytotoxic concentrations of PMO were determined using Alamar Blue in a separate pre-experiment prior to the main study. RAW 264.7 mouse macrophages were seeded in 24-well plates and incubated for 24 h without (untreated) or with lipopolysaccharide (LPS) (100 ng/mL) in absence or presence of different concentrations of PMO (1.25, 2.5, 5, and 10 μg/mL dissolved in 0.5% DMSO, using three independent experiments with two technical replicates each). Supernatants were collected and centrifuged at 10.000×g for 5 min. Tumor necrosis factor (TNF) concentrations in supernatants were determined by ELISA according to the manufacturer’s instruction (Biotechne, United States). Nitric oxide (NO) production in supernatants was measured indirectly via the quantification of nitrite accumulation from NO oxidation processes. Therefore, 100 µL of supernatants were incubated with 100 μL of Griess reagent (Promega, Germany) containing 1% sulfanilamide, 2% phosphoric acid, and 0.1% naphthylethylene diamide. After 20 min of incubation, absorbance was measured at 540 nm. Nitrite concentrations in the cells were determined based on a sodium nitrite standard curve.

### 2.4 Microbiome experiments

To test the effects of PMO on human intestinal microbiota, *in vitro* colonic simulation experiments were conducted using the validated Colon-on-a-Plate® system (ProDigest, Belgium).

#### 2.4.1 Colonic simulation

Fresh human fecal samples were collected from six healthy adults, using the following selection criteria: age between 18 and 50 years, body mass index between 18.5 and 24.9 kg/m^2^, Western diet pattern, not diagnosed with any chronic diseases that could affect the gut microbiome (e.g., diabetes, IBS, inflammatory bowel disease), and had not taken antibiotics during the four months before fecal sample collection. Upon collection, a fecal slurry was prepared from each sample and cryopreserved as described previously (Govaert et al., 2026). The collection and use of fecal samples was conducted as approved by the Ethics Committee of the University Hospital Ghent (reference number ONZ-2022-0267, approved on 29 July 2022). Informed consent was obtained from all subjects involved in the study.

At the start of the colonic simulations, appropriate volumes of PMO were added to the different reactors to achieve the intended final test concentrations of 0.2, 2, and 20 mg/mL. Next, a carbohydrate depleted background medium (PD01, ProDigest, Belgium), representative of the colonic environment, was added and gently sonicated for 10 min to achieve a homogenous dispersion of PMO in the incubation medium. No further surfactants, emulsifiers or additional solvents were used. Prior to addition, the background medium had been boiled to remove oxygen. Finally, cryopreserved fecal inocula from six healthy human donors were added (one donor per reactor; 10% v/v) to provide the microbial communities. Simulation of the mucosal layer was performed by inserting mucus-coated carriers, prepared as described in Van den Abbeele et al., 2012, in all reactors. Each reactor was incubated at 37 °C for 48 h, under continuous mild shaking (90 rpm) and anaerobic atmosphere. Sampling was performed at the start and end of the experiment. For each donor, a No Sample Control (NSC), i.e., a stool sample with background nutritional medium but without addition of any test substance was used as negative control.

Since the samples were incubated in closed systems, overall fermentation activity was measured by monitoring the gas pressure (Δ Gas pressure_T48h-T0h_) using a pressure meter (hand-held pressure indicator CPH6200; Wika, Netherlands).

The tested concentrations were selected to assess potential concentration-dependent effects and to include a concentration estimated from a theoretical luminal exposure based on the recommended daily dose of Digestopret®, a PMO containing herbal medicinal product (3 × 1 capsule, each containing 182 mg PMO). Based on an estimated available colon volume of 250–400 mL (Sender et al., 2016; European Medicines Agency, 2024), the recommended daily dose corresponds to a theoretical luminal concentration range of 1.4–2.2 mg/mL. Therefore, concentrations of 0.2, 2 and 20 mg/mL were included in the colonic simulation experiments to evaluate responses across a broader exposure range.

#### 2.4.2 Microbial community analysis

To best assess the effects of PMO on the microbial community composition, 16S rRNA sequencing was combined with absolute cell quantification using flow cytometry. This enables conversion of relative abundances to absolute counts by multiplying the relative abundances of 16S rRNA sequencing with the total cell count, thereby accounting for different bacterial biomasses (Vandeputte et al., 2017).

##### 2.4.2.1 16S rRNA amplicon sequencing

After DNA extraction as described elsewhere (Perreau et al., 2023), 16S rRNA gene sequencing was conducted using primer set 341F (5′-CCTACGGGNGGCWGCAG-3′) and 785R (5′-GACTACHVGGGTATCTAAKCC-3′), spanning the hypervariable regions V3-V4. Using paired-end sequencing of 2 × 250 bp resulted in amplicons of 424 bp. Bioinformatic processing of the amplicons was done using DADA2 R package (version 4.3.0) according to its tutorial (Callahan et al., 2016). For taxonomic assignment based on SILVA database v138 (Quast et al., 2013) a Naive Bayesian classifier was used. All ASVs classified as Eukaryota, Archaea, Chloroplasts or Mitochondria were removed, as were all ASVs with five or less reads across all samples.

##### 2.4.2.2 Quantification of total bacterial cell counts

Total cell counts were determined using a BD Accuri C6 Plus flow cytometer (BD Biosciences, United States). For sample analysis, the high-flow rate setting with a threshold of 700 on the SYTO channel was used to distinguish cell signals from medium debris and background noise. Proper parent and daughter gates were set to ensure that all bacterial cells were captured.

### 2.5 Statistical analysis

The antispasmodic and anti-inflammatory experiments were conducted as supportive studies to confirm previously reported pharmacological effects of PMO. In contrast, microbiome experiments were designed as exploratory and hypothesis-generating investigations. Therefore, no formal power calculations were conducted, and sample sizes were selected according to established practices and recommendations by the implementing contract research organization.

#### 2.5.1 Antispasmodic and anti-inflammatory experiments

For colonic smooth muscle relaxation, statistical significance was assessed using two-way Analysis of Variance (ANOVA) with Šídák’s comparisons test for comparison of PMO versus solvent effects and one-way ANOVA for comparison of three additions of solvent, respectively. In macrophage cultures, statistical significance was assessed using one-way ANOVA followed by Dunnett’s multiple comparison *post hoc* test. A p-value of ≤ 0.05 was generally considered statistically significant.

#### 2.5.2 Microbiome data

All statistical analyses were performed using R (version 4.5.0; R Core Team, R Foundation for Statistical Computing, Austria). Figures were generated using either R or GraphPad Prism (version 11.0.0 for Windows; GraphPad Software, United States). Microbiome analyses were conducted primarily using the *vegan* (version 2.7.2; Oksanen et al., 2025) and *phyloseq* (version 1.52.0; McMurdie and Holmes, 2013) packages.

##### 2.5.2.1 Absolute abundance calculation

To evaluate the effects of PMO on the microbial community, sequencing reads were processed according to methods described elsewhere (Van den Abbeele et al., 2023). In brief, relative abundances obtained from 16S rRNA gene sequencing were converted to absolute abundances by multiplication with the sample-specific total bacterial cell counts determined by flow cytometry, thereby accounting for differences in biomass across samples.

##### 2.5.2.2 Diversity and fermentation analyses

Changes in total bacterial cell counts, gas production and alpha diversity indices were analyzed using linear mixed-effects models with dose included as a fixed effect and donor included as a random effect to account for inter-individual donor variability. Pairwise comparisons were performed using estimated marginal means with Benjamini–Hochberg adjustment for multiple testing (Benjamini and Hochberg, 1995). Adjusted p-values ≤ 0.05 were considered statistically significant.

To assess sequencing depth coverage, rarefaction curves were generated using the *rarecurve* function implemented in the *vegan* package (Supplementary Figure S3). Alpha diversity analyses were additionally performed on rarefied count data using the minimum sequencing depth observed across all samples (5,521 reads).

##### 2.5.2.3 Community composition and multivariate analyses

To visualize overall community composition, relative genus abundances were displayed as stacked bar charts. For an improved graphical representation, the 15 most abundant genera across all samples were retained individually, whereas the remaining genera were grouped as “Others”.

To assess baseline differences among stool donors prior to incubation, principal component analysis (PCA) was performed on centered log-ratio (CLR) transformed genus-level relative abundances, using the *prcomp* function implemented in R stats package.

To visualize treatment-induced microbial responses independently of baseline differences between donors, the donor-specific log2 fold changes of each genus relative to the corresponding NSC were calculated. PCA was then performed on the resulting log2 fold change matrix from the MaAslin2 analysis. As this analysis was based on log2 fold change values rather than compositional abundance data, a CLR transformation was not applied.

##### 2.5.2.4 Differential abundance analysis

Differential abundance analysis was performed at the genus level using *MaAslin2* package (Mallick et al., 2021) on absolute abundance data derived from 16S rRNA gene sequencing and flow cytometry-based total cell counts. As the primary objective was to assess PMO-specific effects after 48 h of incubation, baseline (0 h) samples were excluded and treatment groups were compared with the corresponding untreated 48 h control (NSC). Prior to analysis, genera present in less than 50% of the samples were removed using a prevalence threshold of 0.5, resulting in 37 genera for downstream analysis. This threshold was chosen to exclude low-prevalence taxa that are more susceptible to zero inflation and unstable effect estimates. Data were analyzed without additional normalization, as absolute rather than relative abundances were used as input. To enable log2 transformation of zero values, a pseudocount of one was added to all abundance values. Dose was included as a fixed effect and donor as a random effect to account for inter-individual donor variability. Multiple-testing correction was performed using Benjamini–Hochberg (Benjamini and Hochberg, 1995) procedure across all genera remaining after prevalence filtering. Genera with q-values ≤ 0.05 were considered significantly differentially abundant.

## 3 Results

### 3.1 PMO was confirmed to have antispasmodic and anti-inflammatory properties

To confirm the antispasmodic properties of PMO, circular smooth muscle strips isolated from the proximal colon of guinea pigs were stimulated *ex vivo* and spontaneous activity was observed in all muscle strips (data not shown). Viability as well as reversibility of the PMO-induced effects were demonstrated in all colonic smooth muscle strips by subsequent potassium chloride (KCl)-induced contractions. Regarding the controls, the non-selective muscarinic agonist arecoline showed a concentration-dependent contraction of colonic smooth muscle strips, while the adenylate cyclase activator forskolin demonstrated concentration-dependent relaxant properties (Supplementary Figures S1A and S1B). The solvent (0.1% DMSO) demonstrated a modest relaxant effect *per se* that remained constant after three repeated additions (−3%, −5% and −6% after the first, second and third additions, respectively; p = 0.6314; Supplementary Figure S1C), whereas PMO induced a significant relaxation of colonic smooth muscle strips at 10 and 100 μg/mL (−13% and −24% decrease compared to the respective DMSO control with p = 0.0281 and p < 0.0001, respectively). The maximal antispasmodic effect was reached for 100 μg/mL (Figure 1).

**FIGURE 1:**
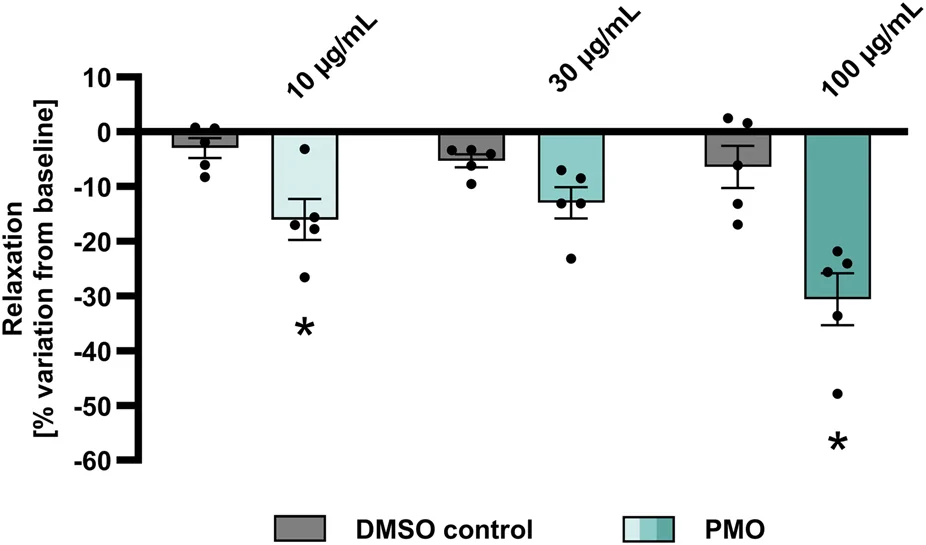
Relaxation of colonic smooth muscle strips induced by PMO. Responses are expressed as % variation from baseline. Data are means ± SEM of n = 5 circular smooth muscle strips of the proximal colon from five guinea pigs. Individual points represent results for single smooth muscle strips. Statistical differences were assessed using two-way ANOVA followed by Šídák´s multiple comparisons test. Significant differences (p ≤ 0.05) compared to the respective DMSO control (solvent) are indicated using asterisks.

To confirm the anti-inflammatory potential of PMO, TNF and NO induction were analyzed *in vitro*. All tested PMO concentrations were not cytotoxic (Supplementary Figure S2). PMO significantly inhibited LPS-induced TNF release in RAW 264.7 mouse macrophages at concentrations of 5 and 10 μg/mL compared to LPS controls, with an inhibition of at least 10% (5 μg/mL PMO: p = 0.0017; 10 μg/mL PMO: p < 0.0001) (Figure 2A). In line with this, NO induction was also inhibited at the same concentrations, reaching a maximum inhibition of approximately 50% at 10 μg/mL (5 μg/mL PMO p < 0.0001; 10 μg/mL PMO: p < 0.0001) (Figure 2B).

**FIGURE 2:**
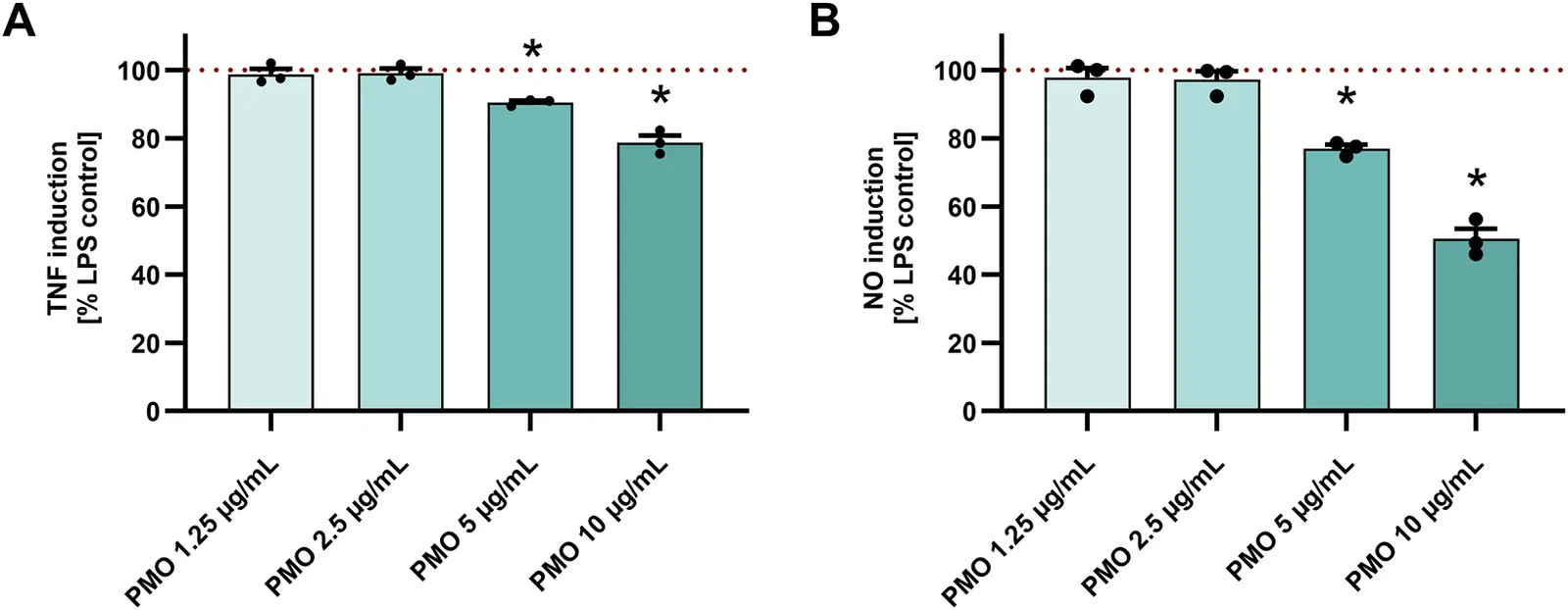
**(A)** TNF secretion and **(B)** NO induction in RAW 264.7 macrophages upon PMO treatment. TNF concentrations were determined by ELISA, and NO production was indirectly assessed using the Griess reagent. Results are based on three independent experiments, each including two technical replicates. Data are presented as mean ± SEM of three independent experiments. Individual points represent means of technical replicates for each independent experiment. Statistical differences were evaluated using two-way ANOVA followed by Dunnett’s multiple comparisons test. Significant differences (p ≤ 0.05) compared to the LPS control (red dotted line) are indicated by asterisks.

### 3.2 Baseline microbiota composition differed between donors

As expected, PCA of the CLR-transformed relative abundances at the genus level revealed differences in microbial community composition among the six donors at the start of the experiment (Figure 3A). Together, the first two principal components (PCs) explained 58% of the total variance. Donor samples were distributed along both PC1 and PC2, with donors A and F separated from the remaining donors along PC1, while the other donors were mainly separated along PC2. The colonic microbiota of the six donors mainly consisted of members of the *Bacillota* (former *Firmicutes*), *Bacteroidota (*former *Bacteroidetes)*, and *Actinomycetota* (former *Actinobacteria*) phyla. Within most donors, the dominant genera within the *Bacillota* phylum were *Subdoligranum, Agathobacter, Blautia and Faecalibacterium*. Within *Bacteroidota* the dominant genus was *Bacteroides*, and within *Actinomycetota* it was *Bifidobacterium* (Figure 3B).

**FIGURE 3:**
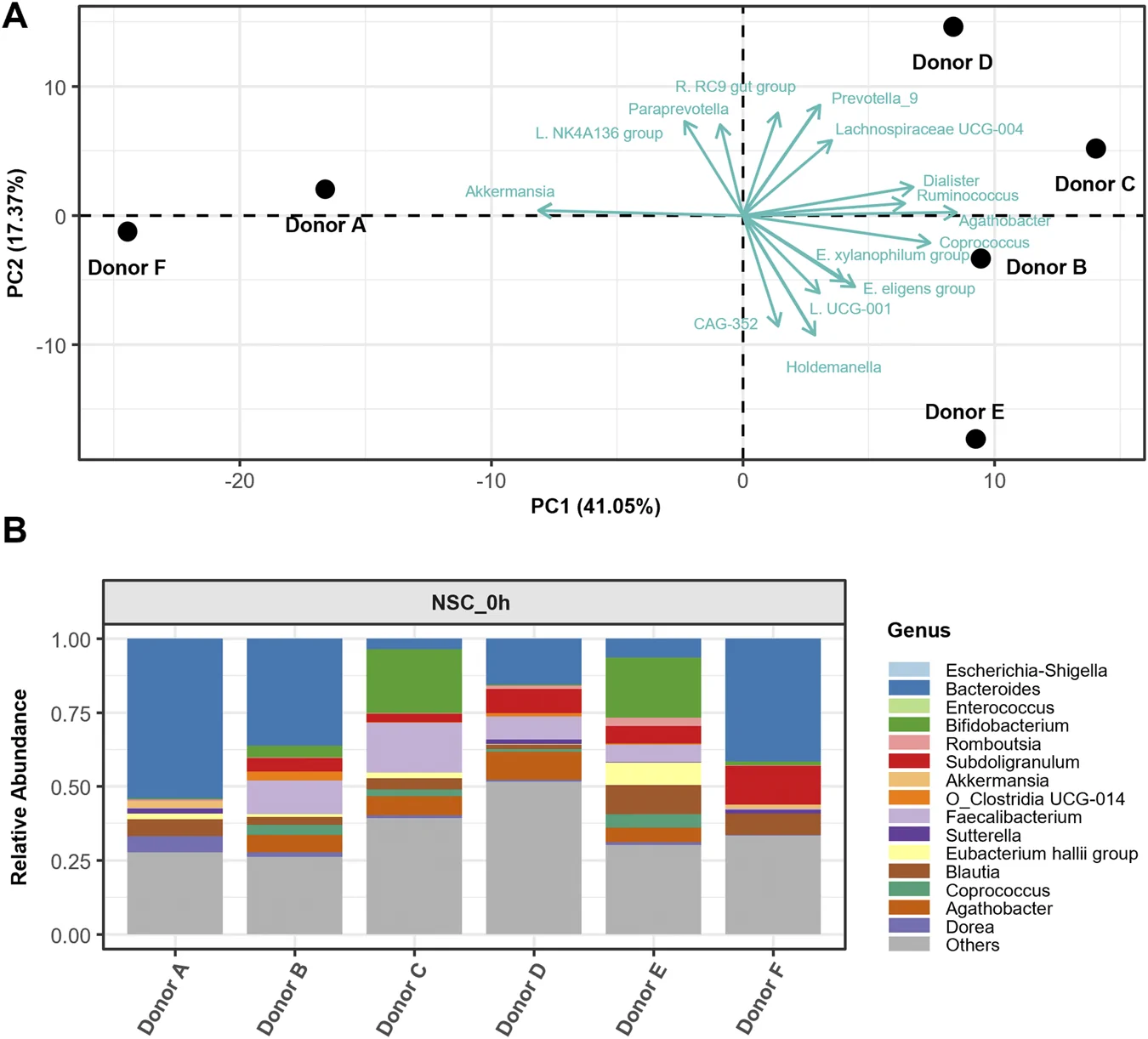
Pronounced donor-specific variation in genus-level microbiota composition at the start of the experiment. Relative abundances of microbiota were assessed on genus level at baseline (n = 6 healthy stool donors, donor A-F using 16S rRNA sequencing. **(A)** PCA of CLR-transformed genus-level relative abundances reveals distinct positioning of donor samples along principal component 1 and 2, indicating variability in microbiota composition across donors. **(B)** Stacked bar plots illustrate differences in genus-level relative abundances between donors. Each bar represents one donor, and colors indicate the 15 most abundant genera. All other genera were grouped as “Others”.

### 3.3 PMO affected total bacterial cell count, gas production and alpha diversity *in vitro*

Total bacterial cell counts were comparable between NSC and the lowest PMO concentration. In contrast, higher PMO concentrations (2 and 20 mg/mL) led to a significant decrease in total bacterial cell counts (both p = 7.84 x 10^−6^). Similarly, gas production remained unchanged at 0.2 mg/mL PMO, but was significantly reduced at PMO concentrations of 2 mg/mL (p = 1.77 x 10^−9^) and 20 mg/mL (p = 1.68 x 10^−9^) (Figure 4A; Supplementary Tables S1 and S2). In contrast, community richness, evenness, and Shannon diversity (Supplementary Table S3) were significantly increased at 2 mg/mL PMO, whereas no significant changes in these indices were observed at 0.2 and 20 mg/mL (Figure 4B). Comparable results were obtained when alpha diversity indices were calculated using rarefied count data (Supplementary Table S4).

**FIGURE 4:**
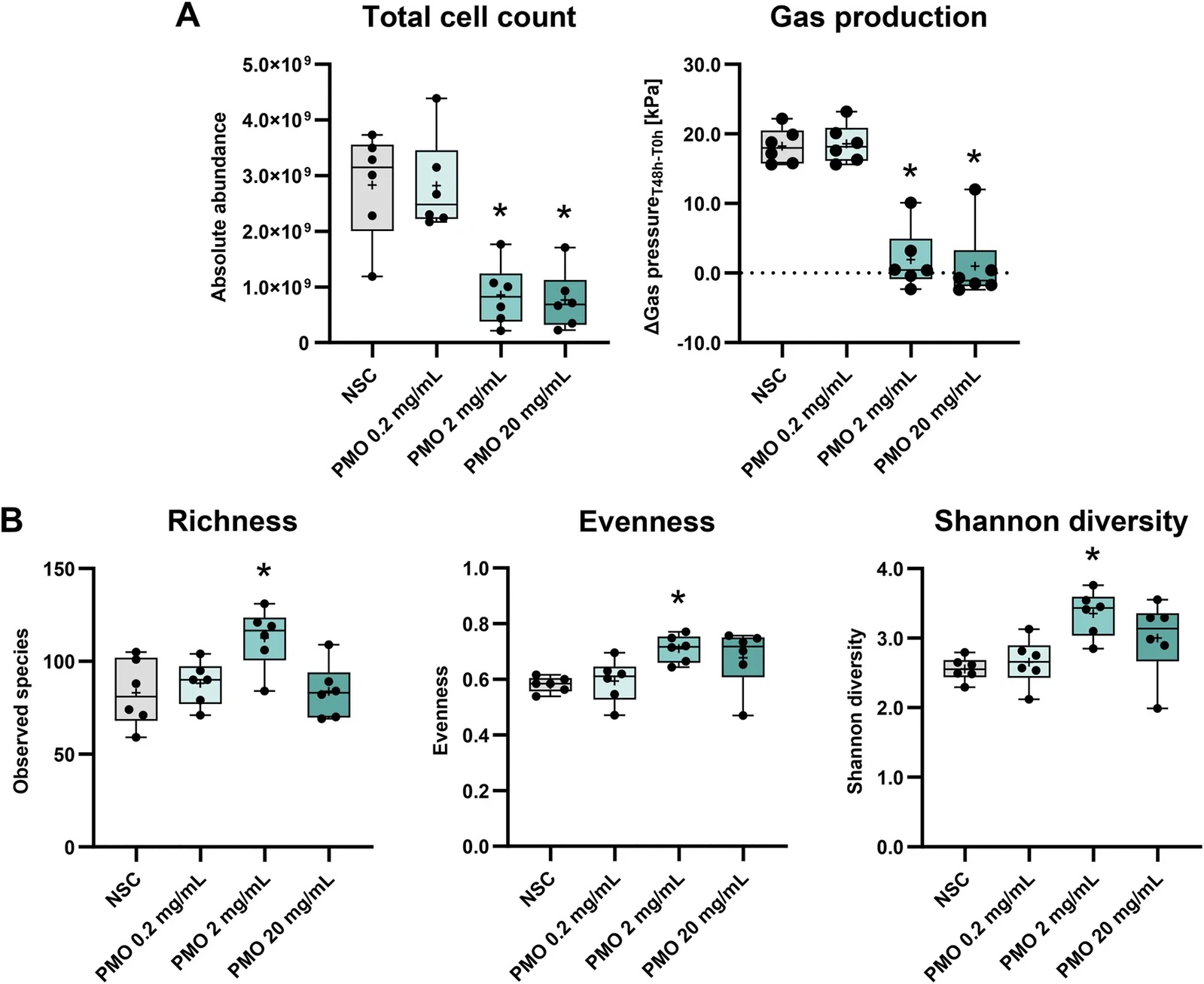
PMO reduced cell count and total gas production, as well as affected different diversity parameters. **(A)** Medium and high concentrations of PMO significantly reduced total cell counts and total gas production after 48 h compared with NSC. **(B)** All alpha diversity metrics (richness, evenness, and Shannon diversity) were increased following treatment with 2 mg/mL PMO and remained stable at 0.2 and 20 mg/mL PMO. Stool samples from n = 6 healthy donors were analyzed and results are depicted as boxplots. Statistical analyses were performed using linear mixed-effects with dose included as a fixed effect and donor included as a random effect. Pairwise comparisons were conducted using estimated marginal means with Benjamini–Hochberg adjustment for multiple testing (Benjamini and Hochberg, 1995). Asterisks indicate significant differences compared with NSC (p < 0.05).

### 3.4 PMO induced concentration-dependent changes in microbial community composition

After 48 h of incubation, bacterial community composition differed between donors under NSC conditions and most PMO treatments (Figure 5). At a PMO concentration of 0.2 mg/mL, bacterial community profiles were similar to those of the corresponding NSC microbiomes after 48 h. In contrast, treatment with 2 and 20 mg/mL PMO resulted in distinct differences in community composition compared to corresponding NSC after 48 h. Under these conditions, gut microbiome profiles were more similar to the corresponding NSCs prior to incubation (0 h, Figure 3B).

**FIGURE 5:**
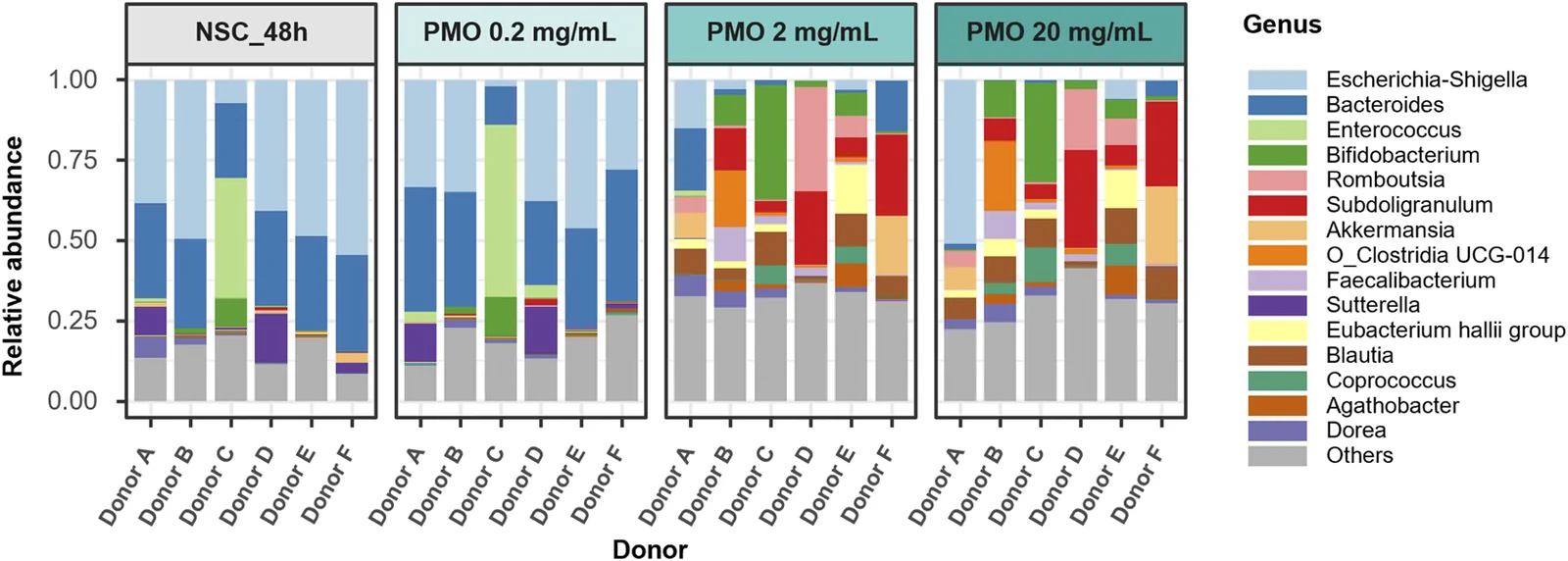
Pronounced donor-specific variation in genus-level microbiota composition following treatment with various concentrations of PMO. Stacked bar plots show the relative abundance of dominant bacterial genera in samples from six individual donors (donors A-F) after 48 h under no substrate control conditions (NSC 48 h) or treatment with increasing concentrations of PMO (0.2, 2, and 20 mg/mL). Each bar represents one donor and colors indicate the 15 most abundant genera, with low-abundance genera grouped as “Others”. Increasing PMO concentrations are associated with marked, donor-dependent shifts in community composition, particularly at 2 and 20 mg/mL.

PMO treatment induced concentration-dependent alterations in microbial community composition, with similar trends observed across the majority of donors (Figure 6). Of the 37 genera remaining after prevalence filtering, 18 showed significant differential abundance for at least one PMO concentration (Supplementary Table S5). For 16 genera, significant changes in abundance were observed at PMO concentrations of 2 and 20 mg/mL, while two genera (*Flavonifractor* and *Gordonibacter*) were significantly affected only at 20 mg/mL PMO. No genus exhibited a significant abundance change at 0.2 mg/mL PMO.

**FIGURE 6:**
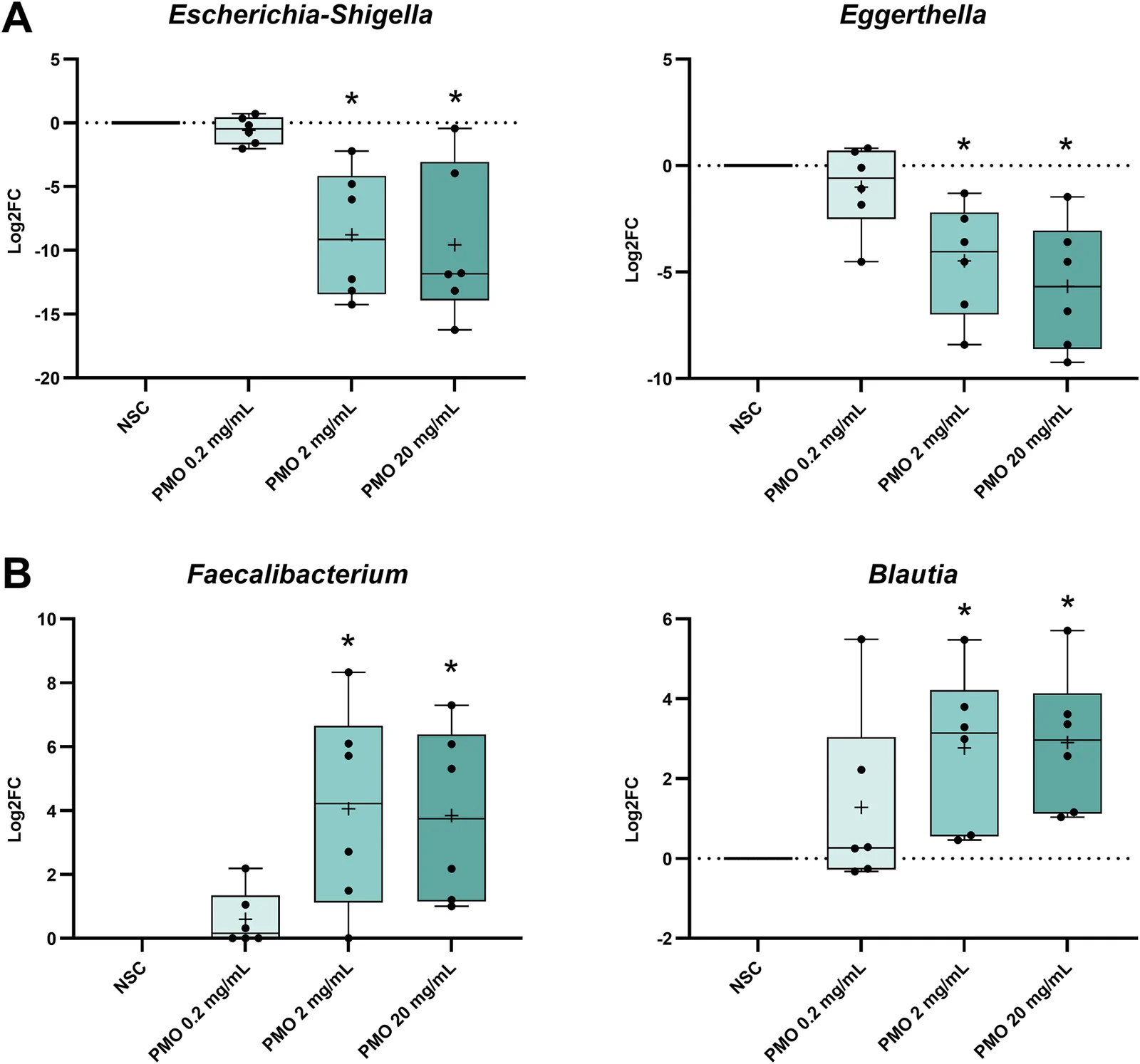
Concentration-dependent alterations in selected genera following PMO treatment. **(A)** Log2 fold changes in absolute abundance of *Escherichia-Shigella* and *Eggerthella* relative to the corresponding donor-specific untreated NSC. **(B)** Corresponding log2 fold changes for *Faecalibacterium* and *Blautia* relative to the corresponding donor-specific untreated NSC. Differential abundance analysis was performed on absolute abundance data using MaAsLin2 with dose as a fixed effect and donor as a random effect. Genera present in fewer than 50% of samples (n = 24) were excluded prior to analysis (prevalence threshold = 0.5). A pseudocount of one was added to each value to enable log transformation. P-values were adjusted for multiple testing using the Benjamini–Hochberg procedure across all tested genera (Benjamini and Hochberg, 1995). Asterisks indicate significant differences compared with NSC (p < 0.05).

*Escherichia-Shigella* and *Eggerthella* showed significantly lower absolute abundances after treatment with 2 and 20 mg/mL PMO compared with the respective donor-specific negative control (NSC), as reflected by negative log2 fold changes (*Escherichia-Shigella*: q = 0.002 and q = 0.001; *Eggerthella*: q = 0.004 and q = 0.001, respectively) (Figure 6A). In contrast, other genera, such as *Faecalibacterium* and *Blautia* were maintained at higher absolute abundances (*Faecalibacterium*: q = 0.007 and q = 0.010; *Blautia*: q = 0.008 and q = 0.006) (Figure 6B). Donor-paired plots of absolute abundance values demonstrated that observed effects were generally consistent across multiple donors and were not solely driven by individual outlying samples (Supplementary Figure S4). The lowest concentration of PMO (0.2 mg/mL) induced only minor and non-significant effects.

To assess overall differences in microbial responses across donors and treatment conditions, PCA based on the genus-level log2 fold change matrix derived from MaAslin2 analysis was performed (Figure 7). The first two PCs accounted for approximately 72% of the total variance in the dataset. Treatment with PMO at concentrations of 2 and 20 mg/mL induced highly similar directional shifts across most donors, whereas samples treated with 0.2 mg/mL PMO clustered separately from the higher PMO concentrations and generally remained closer to the untreated controls.

**FIGURE 7:**
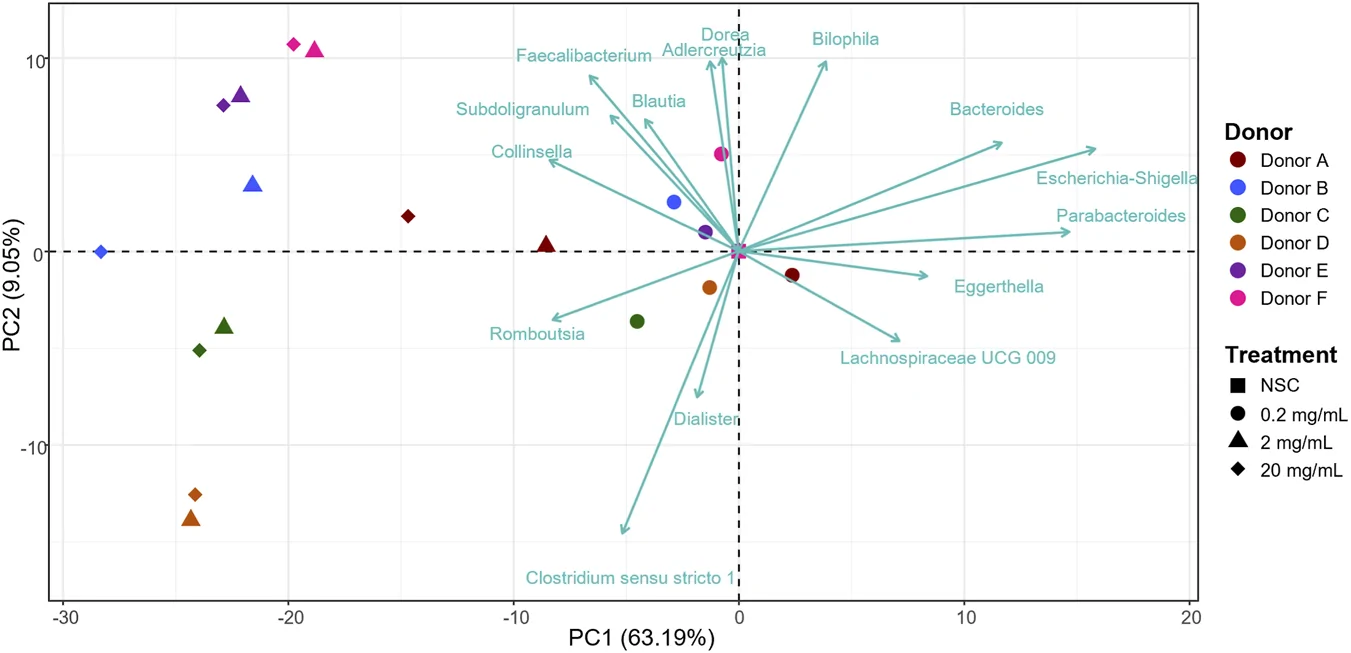
PCA reveals donor-specific clustering and concentration-dependent effects of PMO treatment on microbiota composition with a similar direction of changes across donors. PCA of genus-level microbiota composition across donors (n = 6) and PMO concentrations (none, 0.2, 2, and 20 mg/mL). Points represent individual donor samples, colored by donor identity, and shaped according to PMO concentration. Vectors indicate genera contributing most strongly to community variation. The first two dimensions explained ∼72% of the total variation. In general, increasing PMO concentrations are associated with greater dispersion along the principal components while maintaining pronounced donor-specific clustering.

## 4 Discussion

The reported study assessed several pharmacodynamic effects of PMO that may contribute to its efficacy in the treatment of IBS. While the spasmolytic and anti-inflammatory effects described in literature were confirmed *ex vivo*/*in vitro* for the tested, HMPC monograph-conform PMO (BNO 2910), additional microbiome-related effects were observed *in vitro*. PMO induced concentration-dependent changes in microbial community composition, total bacterial cell counts and reduced gas production in fecal samples obtained from healthy donors. Together, these findings further contribute to elucidating the mode of action of PMO in IBS and suggest that microbiome-associated effects may represent an additional mechanism underlying its overall therapeutic activity.

### 4.1 Confirmation of anti-inflammatory and antispasmodic effects of PMO

PMO exhibited pronounced antispasmodic and anti-inflammatory effects in non-clinical models. At concentrations of 10 and 100 μg/mL, PMO showed a significant relaxation of circular smooth muscles isolated from the proximal colon of guinea pigs. In LPS-stimulated RAW macrophages, PMO exhibited anti-inflammatory effects by significantly inhibiting TNF secretion and NO induction at concentrations of 5 and 10 μg/mL.

The principal effect of PMO in IBS treatment is described as dose-dependent antispasmodic effects on smooth muscles and has been thoroughly investigated previously. This is clearly supported by the reported data. The effect is attributed to Ca^2+^ channel antagonism as observed in animal and human muscle biopsies (Hawthorn et al., 1988; Hills and Aaronson, 1991; Krueger et al., 2020) and is substantiated by clinical evidence showing reduced spasms during colonoscopy after PMO administration (orally or locally in the intestine) (Nehme et al., 2019; Aziz et al., 2020). Besides the antispasmodic properties of PMO, there is evidence for anti-inflammatory effects with relevance to IBS treatment, which were found to be mediated via, among others, the inhibition of the synthesis of pro-inflammatory lipid mediators such as leukotriene B4 (LTB4), prostaglandin E2 (PGE2), and interleukin-1β (IL-1β) as well as TNF secretion (Juergens et al., 1998; Hejna et al., 2021). Furthermore, Al-Mijalli et al. described an inhibitory effect of PMO on 5-lipoxygenase with a half maximal inhibitory concentration (IC_50_) of 11.64 μg/mL (Al-Mijalli et al., 2022) comparable to the highest concentration used to treat LPS-stimulated RAW macrophages in the presented study. Moreover, increased NO levels were observed in colon tissue from patients with IBS (Koçak et al., 2016), supporting the clinical relevance of the observed *in vitro* dose-dependent reduction of NO levels by PMO.

The anti-inflammatory and antispasmodic effects of encapsulated PMO shown in non-clinical models in the present study underscore the therapeutic potential of PMO for alleviating symptoms of IBS. While the antispasmodic effects are suggested to be the principal mechanism for amelioration of gastrointestinal spasm-associated abdominal pain (Capanni et al., 2005; Cappello et al., 2007), the anti-inflammatory effects of PMO might contribute to its beneficial effect since there is evidence that IBS is associated with low-grade intestinal inflammation (Holtmann et al., 2016; Meynier et al., 2022).

### 4.2 Impact of PMO on the human gut microbiome and its potential clinical implications

In addition to the previously reported and in the present study confirmed antispasmodic and anti-inflammatory effects, PMO also induced marked changes in microbial community composition and gas production *in vitro* in human stool samples obtained from healthy donors. In particular, higher PMO concentrations were associated with marked reductions in total bacterial cell counts and gas production, accompanied by alterations in microbial community composition. Despite these changes, alpha diversity was largely preserved across treatment conditions. Together, these findings expand the current understanding of the multifactorial mode of action of PMO and highlight the potential relevance of microbiome-associated effects alongside its established antispasmodic and anti-inflammatory properties.

#### 4.2.1 Effects of PMO on microbial diversity

Preservation of microbial diversity is widely regarded as an indicator of resilience and functional stability of the gut microbiome as a complex ecosystem (Lozupone et al., 2012; Sommer et al., 2017; Safarchi et al., 2025). In the present study, alpha diversity was largely preserved across PMO concentrations despite substantial reductions in bacterial cell counts and gas production. The observed increase in evenness at a PMO concentration of 2 mg/mL indicates a more balanced distribution of community members and a reduced dominance of individual taxa. These characteristics are commonly associated with ecosystem stability in microbial communities. Nevertheless, the functional consequences of these changes cannot be inferred directly from diversity metrics alone. In subsets of IBS patients the microbial diversity has been reported to be reduced (Barandouzi et al., 2021; Shaikh et al., 2023) and therefore preservation or even a slight increase of microbial diversity would be considered a favorable characteristic but warrants disease-specific studies.

#### 4.2.2 Effects of PMO on microbial community composition

In addition to the observed diversity-related effects, PMO induced concentration-dependent alterations in microbial community composition compared with the respective untreated controls. At PMO concentrations of 2 and 20 mg/mL, *Escherichia-Shigella* and *Eggerthella* exhibited lower absolute abundances. Both genera have been associated with dysbiosis and inflammation-related microbial profiles (Hu et al., 2022; Goudman et al., 2024).

The *Escherichia-Shigella* group includes pathogens that are frequently increased under inflammatory conditions and contribute to epithelial barrier dysfunction and gut immune responses (Wang et al., 2020). Additionally, increased levels of *Pseudomonadota* (former *Proteobacteria*, including *Escherichia-Shigella*) are widely regarded as a hallmark of gut microbiome dysbiosis associated with increased inflammatory signals (Shin et al., 2015; Cheng et al., 2024). Notably, Hu et al. reported elevated abundances of this genus together with reduced abundances of SCFA-producing bacteria in dysbiosis-associated microbial communities, suggesting that expansion of this genus may occur as part of broader microbiome alterations rather than as an isolated taxonomic change (Hu et al., 2022).

*Eggerthella* spp. have been associated with multiple diseases and systemic dysfunctions (Dong et al., 2022; Goudman et al., 2024; Abahussin et al., 2025; Shin et al., 2025). Notably, *Eggerthella lenta*, the best characterized species within this genus, has been found to be involved in diverse metabolic processes, including the transformation of host-derived and xenobiotic compounds, such as bile acids and pharmaceutical agents (Haiser et al., 2013) and the production of bioactive metabolites that directly modulate host immune responses (Shin et al., 2025). These metabolic activities are particularly relevant in the context of IBS, where altered bile acid metabolism, dysregulated microbial metabolite production, and visceral hypersensitivity are recognized contributors to disease pathophysiology (Xiao et al., 2021; Zhan et al., 2024; Aggeletopoulou et al., 2025; Li et al., 2025).

Taken together, the observed reduction of absolute abundances of *Escherichia-Shigella* and *Eggerthella* demonstrates that PMO counteracted the expansion of these genera under the experimental conditions. While the present study does not allow to draw direct conclusions regarding the correction of IBS-associated dysbiosis *in vivo*, these observations are noteworthy given previous reports linking both genera to inflammation-associated and dysbiosis-related microbial profiles.

In contrast to the reductions of *Escherichia-Shigella* and *Eggerthella*, PMO maintained higher abundances of genera such as *Faecalibacterium* and *Blautia* than observed in the corresponding NSCs. Reduced levels of *Faecalibacterium* are a consistent feature of IBS and inflammatory bowel disease and have been shown to negatively correlate with disease severity (Rajilić-Stojanović et al., 2011; Chen et al., 2023; Martin et al., 2023). *Faecalibacterium*, particularly *Faecalibacterium prausnitzii*, is widely recognized for its anti-inflammatory properties and its role in maintaining epithelial barrier integrity (Sokol et al., 2008; Miquel et al., 2013; Danne et al., 2026). For example, therapeutic application of *Faecalibacterium prausnitzii*, *Bacteroides faecis* and *Roseburia intestinalis* (alone and in combination) in mice significantly ameliorated DSS-induced colitis in terms of clinical symptoms, histological inflammation, and immune status (Mohebali et al., 2023), highlighting the potential relevance of microbial community composition for host inflammatory responses.

*Blautia* represents a dominant intestinal genus with documented immunomodulatory functions, including suppression of pro-inflammatory cytokines, regulation of intestinal T-cell responses, and contributions to SCFA-associated metabolic pathways (Liu et al., 2021; Wei et al., 2025). While genus-level associations may vary between disease entities, species-level analyses suggest protective and anti-inflammatory roles for specific *Blautia* species, such as *Blautia luti* and *Blautia wexlerae* (Benitez-Paez et al., 2020).

Importantly, as the present study combined 16S rRNA gene sequencing with flow cytometry-based cell quantification, the observed changes reflect alterations in absolute rather than only relative abundances. Consequently, the reduced abundances of *Escherichia-Shigella* and *Eggerthella*, as well as the maintenance of higher abundances of *Faecalibacterium* and *Blautia*, cannot be explained solely by compositional effects resulting from changes in total bacterial biomass. This distinction is particularly relevant given the pronounced reduction in total bacterial cell counts observed at higher PMO concentrations.

Our findings are broadly consistent with observations by Lehner et al., who reported preserved alpha diversity and treatment-associated alterations in microbial community composition following *in vitro* incubation of human gut microbiota obtained from healthy stool donors with a combination of PMO and caraway oil (Lehner et al., 2025). In that study, relative abundances of beneficial, SCFA-associated genera (e.g., *Bifidobacterium*) increased, while inflammation-associated genera, such as *Bacteroides* and *Parabacteroides,* were reduced. Similar microbial shifts were observed for PMO alone.

However, important methodological differences limit direct comparability of the two studies, such as the absence of absolute abundance measurements and shorter incubation times in the study by Lehner et al. Moreover, this includes the use of a combination product, leading to a lower PMO exposure. Additionally, only a single concentration of the PMO–caraway oil combination was investigated, which limits interpretation with respect to dose–response relationships and does not allow conclusions on concentration-dependent microbiome effects. Nevertheless, the findings of both studies suggest that PMO alone is capable of inducing microbiome-associated effects *in vitro*.

Evidence from *in vitro* studies with fecal samples from IBS patients, *in vivo* studies and clinical trials, however, is lacking. To the best of our knowledge, only a single clinical trial by Thapa et al. investigated the specific effects of PMO on the human gut microbiome (Thapa et al., 2022). Children with functional abdominal pain were treated with PMO (180, 360, or 540 mg per day). The authors found no significant change in the overall composition of the human gut microbiome after treatment with PMO for 1 week (Thapa et al., 2022). However, there was a significant change in the abundance of some bacteria (e.g., *Collinsella)*. Thapa et al. hypothesized that a higher dose and/or longer duration of treatment with PMO might be worth investigating to support the microbiome-associated effects of PMO.

All in all, the present study demonstrates that PMO induces concentration-dependent microbiome-associated effects *in vitro*, including alterations in microbial community composition while largely preserving microbial diversity. Whether these microbiome-associated changes contribute to the clinical efficacy of PMO in IBS remains to be established in IBS-specific models and clinical trials. Nevertheless, the observed effects suggest that microbiome-associated mechanisms warrant further investigation as potential contributors to the overall pharmacological profile of PMO.

#### 4.2.3 Concentration-dependent microbial responses and donor-specific variability

The tested concentration range of PMO included a theoretical luminal exposure estimate derived from the recommended daily PMO dose and was extended by a 10-fold lower and higher concentration to assess concentration-dependent effects. At the lowest tested concentration (0.2 mg/mL), PMO had negligible effects on total bacterial cell counts and total gas production. In contrast, both 2 and 20 mg/mL induced pronounced microbiome-associated effects, including reductions in total bacterial abundance, gas production and concentration-dependent alterations in microbial community composition. The highly similar responses observed at 2 and 20 mg/mL PMO suggest that the maximal microbiome response may already have been reached at approximately 2 mg/mL. Given the established contribution of excessive intestinal gas formation to symptoms such as bloating, abdominal discomfort, and pain, the observed reduction in gas production may be of particular interest in the context of IBS (Crucillà et al., 2024). At the same time, the biological significance of this response is likely context-dependent and should be considered alongside the broader changes in microbial abundance and activity, as well as community composition observed following PMO treatment.

Despite pronounced inter-individual differences in baseline microbiota composition, the direction of PMO-induced changes was largely consistent across donors. Nevertheless, multivariate analyses demonstrated stable donor-specific clustering, indicating that initial community structure remained a major determinant of the magnitude and specific pattern of PMO-induced effects as previously observed in studies with dietary interventions (Korpela et al., 2014; Kolodziejczyk et al., 2019) or probiotic or prebiotic treatment (Griffin et al., 2017; Zmora et al., 2018).

Importantly, microbial communities in samples treated with 2 and 20 mg/mL of PMO clustered closer to baseline (0 h) profiles than to untreated 48 h controls. This observation suggests that PMO partially mitigated incubation-associated shifts in community composition while preserving donor-specific community characteristics. The persistence of donor-specific clustering indicates that PMO did not override baseline inter-individual microbiota differences but rather modulated community composition within the context of each donor microbiome composition. Such behavior is characteristic for complex ecosystems and is consistent with previous observations regarding microbiota resilience (Rajilić-Stojanović et al., 2011).

Taken together, the reported data support a multimodal mode of action of PMO that may explain its clinical efficacy in IBS. Alongside its established antispasmodic and anti-inflammatory activities, the present *ex vivo* study showed additional microbiome-associated effects, including concentration-dependent alterations on microbial community composition, as well as reduced bacterial cell counts and gas production in stool samples from healthy donors. Although the clinical relevance of these microbiome-associated findings remains to be established, they may represent relevant effects contributing to the overall positive pharmacological profile of PMO. An influence on the IBS-associated symptoms of bloating and abdominal discomfort and pain therefore appears very likely. Altogether, the well-known clinical benefit of PMO in treating patients suffering from IBS is likely attributable to the described as well as additional effects not further discussed in the present publication, such as, e.g., TRPM8-mediated afferent desensitization (Harrington et al., 2011).

## 5 Study limitations and translational considerations

The present study included both supportive pharmacological experiments and exploratory microbiome analyses. As the antispasmodic and anti-inflammatory effects of PMO are already well established and were investigated primarily to confirm previous findings, the following limitations and considerations mainly relate to the microbiome experiments of the study.

The microbiome experiments were conducted using an *in vitro* fermentation system, a relatively short incubation period (48 h) and stool samples obtained exclusively from healthy donors. While this design allows controlled investigation of direct short-term microbiome-associated effects of PMO, long-term dynamics and disease-specific responses cannot be directly inferred. In addition, *in vitro* fermentation systems cannot fully reflect important physiological aspects of the gastrointestinal tract, such as continuous nutrient flow, absorption processes, formulation-dependent release characteristics or spatial variation along the intestine. These factors may influence the local exposure of the microbiota to PMO and its metabolites. Nevertheless, an initial clinical trial with patients with IBS-like symptoms (Thapa et al., 2022) found no association of short-term PMO administration with major changes in overall gut microbiome composition. Finally, the microbiome experiments were based on a relatively small number of donors and should therefore be regarded as exploratory and hypothesis-generating. Validation in larger donor cohorts, IBS-specific models and clinical trials is required. This is particularly relevant given the pronounced inter-individual variability of the gut microbiome and its known influence on treatment responses.

## 6 Conclusion

PMO (BNO 2910) exhibited pronounced antispasmodic and anti-inflammatory effects in non-clinical models, substantiating its use for the management of IBS-related symptoms, such as abdominal pain/distension. In addition, PMO induced concentration-dependent microbiome-associated effects in samples from healthy donors, including alterations in microbial community composition, preservation of microbial diversity, and reduced gas production. Together, these findings provide additional insight into the multimodal pharmacology of PMO. While the clinical relevance of the observed microbiome-associated effects remains to be established, the reduction in gas production may be of particular interest in the context of IBS, where bloating and abdominal discomfort/pain represent important symptom burdens. Future clinical studies should investigate these observations in patients suffering from IBS.

## Data Availability

The raw sequence files associated with this study have been deposited in the NCBI Sequence Read 545 Archive (SRA) and can be accessed through BioProject accession PRJNA1526452.
