# Supplementary material for: Peppermint oil exhibits antispasmodic and anti-inflammatory properties and alters gut microbial community composition and gas production in vitro
Source: Front Pharmacol. 2026 Oct 22;17:1963380. doi: 10.3389/fphar.2026.1963380 (PMC13736052; doi:10.3389/fphar.2026.1963380)
Supplement: Supplementary file 1 [file DataSheet1.pdf]

Supplementary material

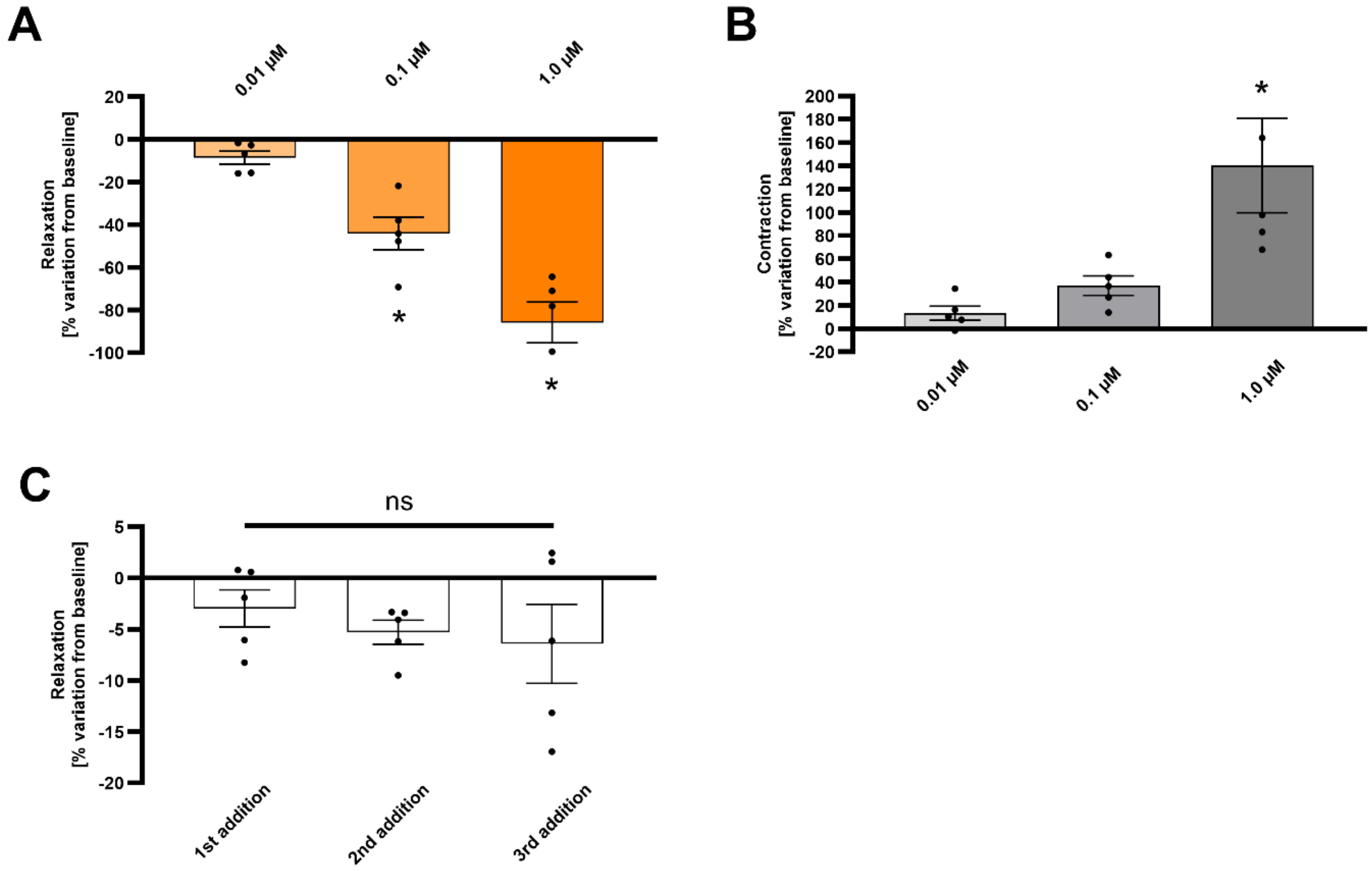

Supplementary Figure S1. Effects of controls on colonic smooth muscle strips from guinea pigs.

Responses are expressed as % variation from baseline. Data are means ± SEM of n = 5 circular smooth muscle strips from the proximal colon of five guinea pigs. Individual points represent results for single smooth muscle strips (one datapoint each for forskolin and arecoline at 1.0 µM are outside the axis limits). (**A**) Relaxation of colonic smooth muscle strips induced by forskolin. (**B**) Contraction of colonic smooth muscle strips induced by arecoline. Statistical differences were assessed using two-way ANOVA followed by Šídák's multiple comparisons test (forskolin and arecoline). Significant differences ($p \leq 0.05$) compared to the respective DMSO control (solvent) are indicated using asterisks. (**C**) Effects of solvent (containing 0.1 % DMSO). Statistical differences were assessed using one-way ANOVA. ns: not significant.

Determination of cytotoxicity in RAW 264.7 macrophages

Cell cultures were stimulated with selected concentrations of PMO (4 concentrations, n = 2) for 24 h. Cell viability was determined by Alamar Blue staining using NaF (100, 250, and 500 µg/mL) as toxic control. Alamar Blue (ThermoFisher Scientific, USA) is a redox indicator that yields a colorimetric change and a fluorescent signal in response to metabolic activity. The colorimetric reaction was determined by an ELISA reader measuring the intensity of the red color as marker of the increased Alamar Blue reaction. The decrease of metabolism/cell viability was expressed in comparison to untreated control cells. Effects significantly higher than a 25 % decrease in this assay were concluded to be due to cell toxic effects or slowing down cell metabolism (< 75 % viability), according to the acceptance criteria of the contract research organization.

Based on the results of the two experiments shown below (Supplementary Figure S2), 10 µg/mL PMO was selected as highest concentration for assessing TNF secretion and NO induction in RAW 267.4 macrophages.

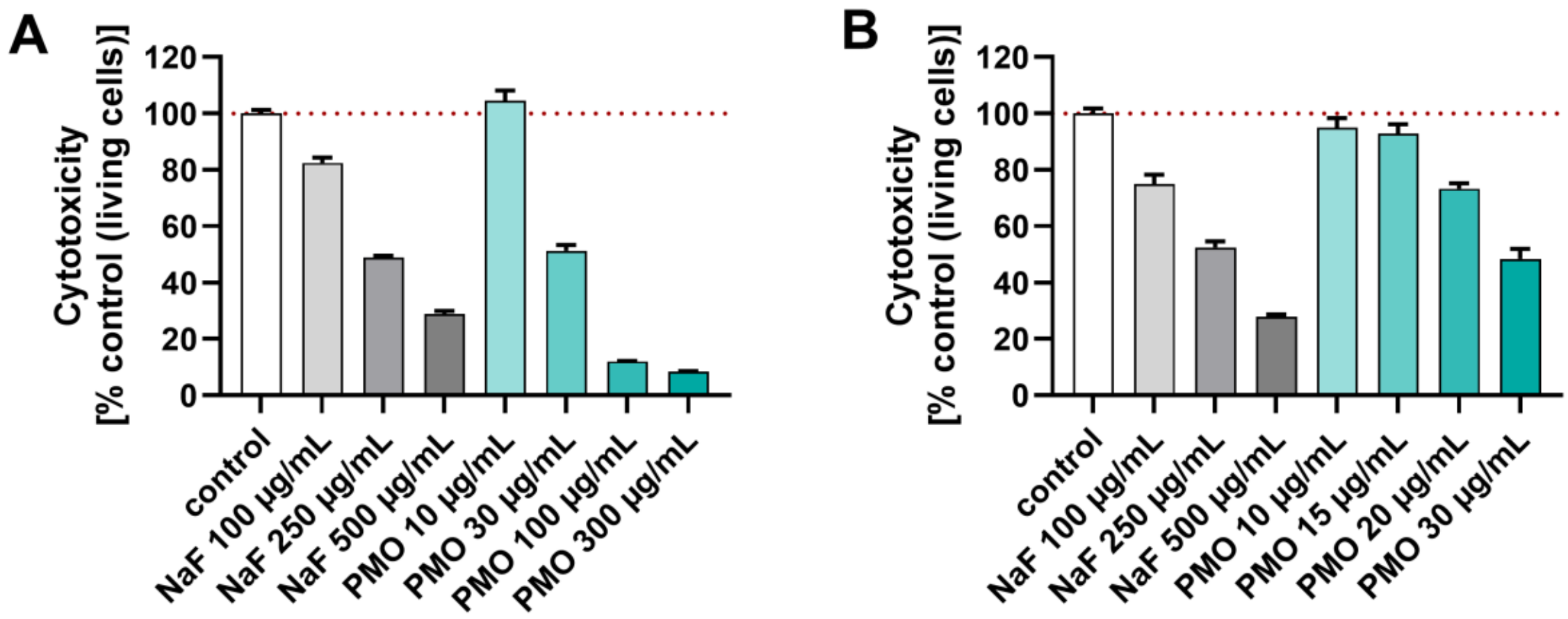

Supplementary Figure S2. Cytotoxic effects of PMO in RAW 264.7 macrophages.

Cytotoxicity was assessed by ELISA using Alamar Blue as a redox indicator. **(A)** In the first experiment the concentration range of 10 to 300 µg/mL PMO was tested. **(B)** The second experiment focused on the relevant concentration range of 10 to 30 µg/mL PMO. Data are presented as mean ± SEM of technical duplicates (PMO) and quadruplicates (control and NaF), respectively.

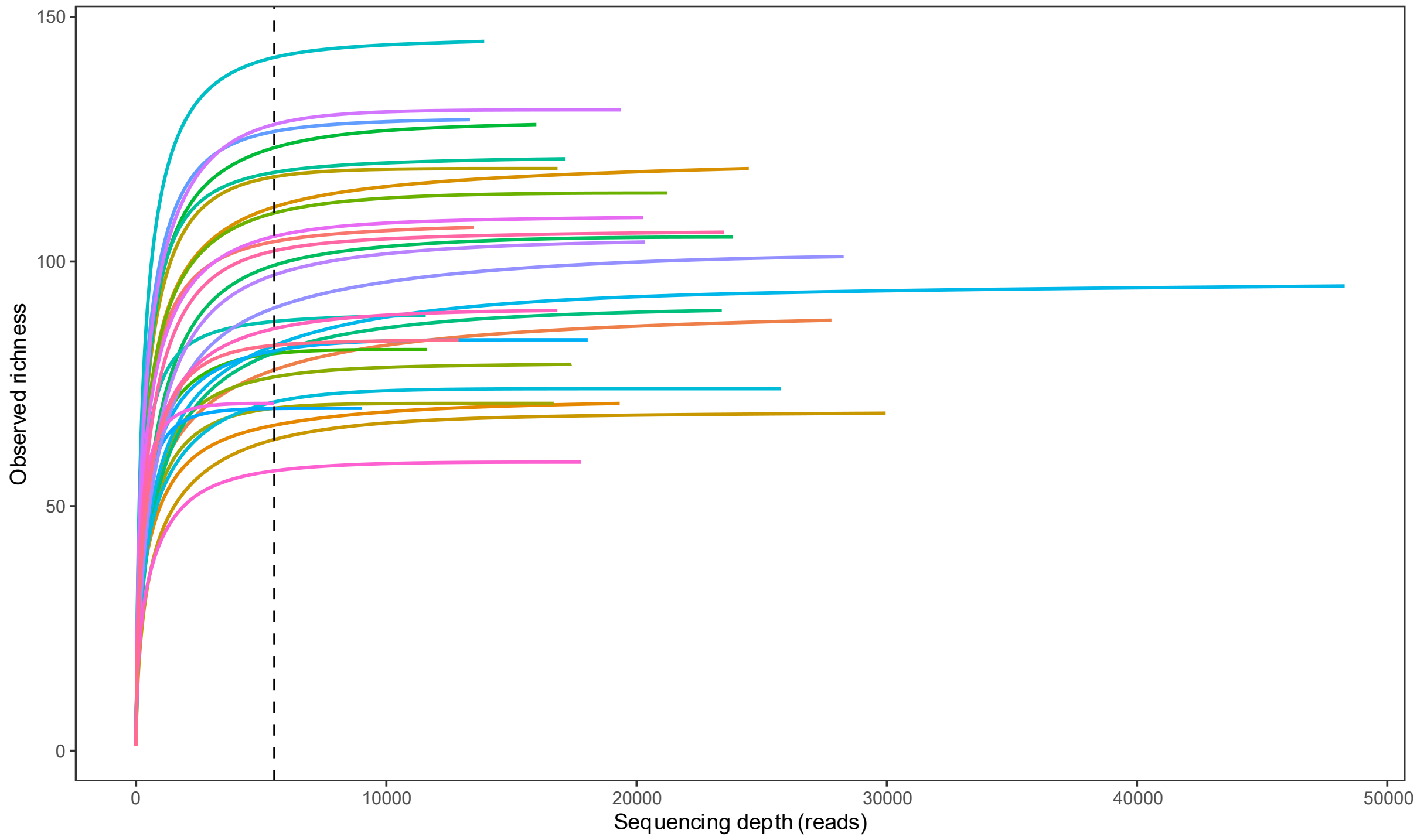

Supplementary Figure S3. Rarefaction curves for all samples included in the colonic simulation experiments.
Rarefaction curves (n = 30) were generated from ASV-level sequencing data to assess whether sequencing depth was sufficient to capture the majority of the observed microbial diversity. The vertical dashed line indicates the rarefaction depth used for subsequent diversity analyses and corresponds to the minimum sequencing depth observed across all samples. Despite the relatively low rarefaction depth of 5,521 reads, most curves approached a plateau before reaching this threshold, suggesting that sequencing depth was sufficient to recover most of the observable bacterial diversity.

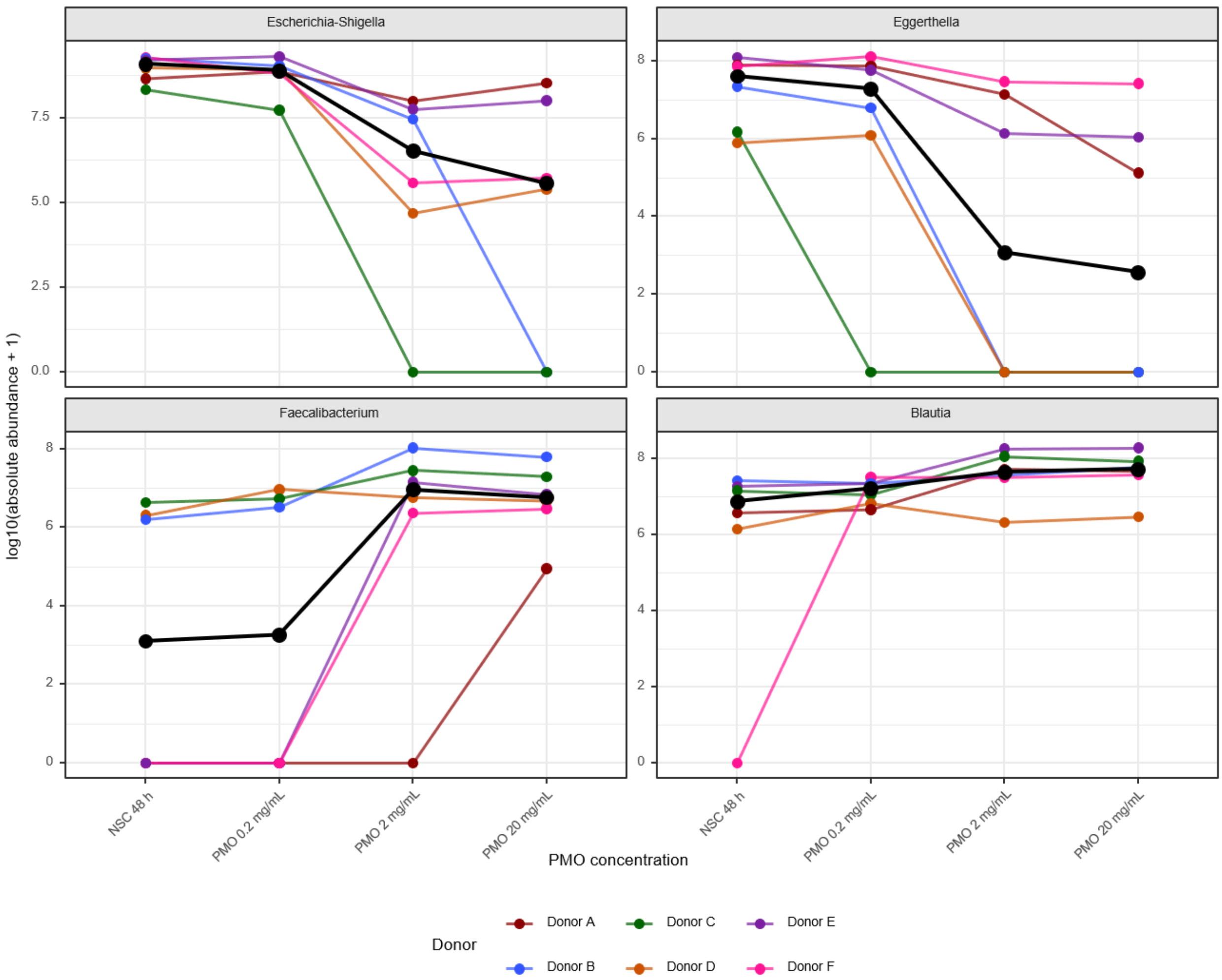

Supplementary Figure S4. Donor-specific paired plots of absolute abundances for selected genera identified by differential abundance analysis.
Lines connect samples originating from the same donor across the untreated 48 h control (NSC) and the different PMO concentrations (0.2, 2, and 20 mg/mL). Abundances are shown as log10-transformed absolute abundances derived from 16S rRNA gene sequencing and flow cytometry-based total bacterial cell counts. Prior to log10-transformation, a pseudocount of 1 was added to all abundance values. Connected data points represent the different treatment conditions for each donor (n = 6). Genera shown correspond to key taxa identified by the MaAsLin2 differential abundance analysis.

Supplementary Table S1. Total bacterial cell counts and change in gas pressure for all samples.
Total bacterial cell counts were quantified by flow cytometry and gas production was assessed as the change in headspace pressure (Δ gas pressure) during the 48 h incubation period.

Donor	Timepoint	Dose	Δ Gas pressure$_{Sample-T0h}$	Cell Count
A	0 h	NSC	0	2.74E+08
A	48 h	NSC	17.2	1.19E+09
A	48 h	0.2 mg/mL	15.6	2.24E+09
A	48 h	2 mg/mL	0.5	6.45E+08
A	48 h	20 mg/mL	-1.5	6.65E+08
B	0 h	NSC	0	1.45E+09
B	48 h	NSC	15.6	3.73E+09
B	48 h	0.2 mg/mL	18.7	3.15E+09
B	48 h	2 mg/mL	3.2	1.01E+09
B	48 h	20 mg/mL	-1.7	7.11E+08
C	0 h	NSC	0	1.86E+09
C	48 h	NSC	15.8	3.01E+09
C	48 h	0.2 mg/mL	16.3	2.67E+09
C	48 h	2 mg/mL	-0.4	1.07E+09
C	48 h	20 mg/mL	-0.7	9.32E+08
D	0 h	NSC	0	3.49E+08
D	48 h	NSC	18.8	2.28E+09
D	48 h	0.2 mg/mL	17.6	2.17E+09
D	48 h	2 mg/mL	-2.3	2.15E+08
D	48 h	20 mg/mL	-2.4	2.24E+08
E	0 h	NSC	0	1.53E+09
E	48 h	NSC	22.2	3.29E+09
E	48 h	0.2 mg/mL	23.2	4.39E+09
E	48 h	2 mg/mL	10.1	1.77E+09
E	48 h	20 mg/mL	12	1.71E+09
F	0 h	NSC	0	5.16E+08
F	48 h	NSC	19.9	3.50E+09
F	48 h	0.2 mg/mL	20.1	2.30E+09
F	48 h	2 mg/mL	0.4	4.36E+08
F	48 h	20 mg/mL	0.4	3.49E+08

Supplementary Table S2. Results of linear mixed-effects models for total bacterial cell counts and gas production.

Statistical significance was evaluated using linear mixed-effects models with dose (n = 4, including NSC) included as a fixed effect and donor (n = 6) included as a random effect. Pairwise comparisons were performed using estimated marginal means with Benjamini-Hochberg adjustment for multiple testing. Effect estimates, corresponding 95 % confidence intervals, and adjusted p-values are shown.

Endpoint	Contrast	Estimate	Lower 95% CI	Upper 95% CI	Adjusted p-value
Cell Count	NSC - PMO 0.2 mg/mL	1.54E+07	-8.50E+08	8.81E+08	9.58E-01
Cell Count	NSC - PMO 2 mg/mL	1.98E+09	1.11E+09	2.84E+09	7.84E-06
Cell Count	NSC - PMO 20 mg/mL	2.07E+09	1.20E+09	2.93E+09	7.84E-06
Gas production	NSC - PMO 0.2 mg/mL	-0.333	-4.094	3.428	7.92E-01
Gas production	NSC - PMO 2 mg/mL	16.333	12.572	20.094	1.77E-09
Gas production	NSC - PMO 20 mg/mL	17.233	13.472	20.994	1.68E-09

Supplementary Table S3. Summary of results for alpha diversity calculations on relative abundances and absolute raw counts.

Alpha diversity was assessed using Shannon diversity, Pielou's evenness, and observed richness. Calculations were conducted for relative abundance data, as well as rarefied absolute raw counts. Sequencing depth used for rarefaction was the lowest overall value over all samples.

Donor	Timepoint	Dose	Raw counts	Relative abundance data			Rarefied raw counts		
				Shannon diversity	Pielou's evenness	Observed richness	Shannon diversity	Pielou's evenness	Observed richness
A	0 h	NSC	13,487	3.57	0.76	107	3.57	0.77	106
A	48 h	NSC	27,788	2.62	0.58	88	2.61	0.60	78
A	48 h	0.2 mg/mL	19,324	2.57	0.6	71	2.58	0.61	67
A	48 h	2 mg/mL	24,483	3.41	0.71	119	3.42	0.73	111
A	48 h	20 mg/mL	29,953	1.99	0.47	69	2.00	0.48	64
B	0 h	NSC	16,840	3.65	0.76	119	3.64	0.76	117
B	48 h	NSC	16,687	2.49	0.58	71	2.46	0.58	69
B	48 h	0.2 mg/mL	17,395	2.75	0.63	79	2.73	0.63	75
B	48 h	2 mg/mL	21,213	3.54	0.75	114	3.54	0.75	109
B	48 h	20 mg/mL	11,607	3.29	0.75	82	3.30	0.75	81
C	0 h	NSC	15,996	3.52	0.73	128	3.51	0.73	124
C	48 h	NSC	23,846	2.79	0.6	105	2.78	0.61	97
C	48 h	0.2 mg/mL	23,404	2.12	0.47	90	2.10	0.48	78
C	48 h	2 mg/mL	17,138	3.45	0.72	121	3.48	0.73	120
C	48 h	20 mg/mL	11,573	3.29	0.73	89	3.29	0.74	87
D	0 h	NSC	13,910	4.00	0.8	145	4.00	0.81	143
D	48 h	NSC	25,759	2.65	0.62	74	2.63	0.62	72
D	48 h	0.2 mg/mL	48,297	2.82	0.62	95	2.80	0.64	81
D	48 h	2 mg/mL	18,047	2.85	0.64	84	2.89	0.65	84
D	48 h	20 mg/mL	9,025	2.98	0.7	70	2.99	0.70	70
E	0 h	NSC	13340	3.79	0.78	129	3.78	0.78	127
E	48 h	NSC	28,276	2.49	0.54	101	2.48	0.55	92
E	48 h	0.2 mg/mL	20,323	2.54	0.55	104	2.53	0.56	96
E	48 h	2 mg/mL	19,372	3.76	0.77	131	3.74	0.77	130
E	48 h	20 mg/mL	20,271	3.55	0.76	109	3.56	0.76	105
F	0 h	NSC	5,521	3.42	0.8	71	3.42	0.80	71
F	48 h	NSC	17,767	2.30	0.56	59	2.31	0.57	57
F	48 h	0.2 mg/mL	16,835	3.13	0.7	90	3.12	0.70	87
F	48 h	2 mg/mL	23,504	3.10	0.66	106	3.07	0.67	100
F	48 h	20 mg/mL	12,887	2.90	0.65	84	2.88	0.65	82

Supplementary Table S4. Results of alpha diversity analyses based on original and rarefied raw counts

Alpha diversity was assessed using Shannon diversity, Pielou's evenness, and observed richness. Metrics were calculated from both the original dataset and a rarefied dataset generated at the minimum sequencing depth observed across all samples (5,521 reads). Statistical analyses were performed using linear mixed-effects models with dose (n = 4, including NSC) included as a fixed effect and donor included as a random effect. Pairwise comparisons were conducted using estimated marginal means with Benjamini-Hochberg adjustment for multiple testing. Effect estimates, corresponding 95 % confidence intervals (CI), and adjusted p-values are reported. Negative estimates indicate higher alpha diversity values in the PMO-treated group relative to the untreated 48 h control (NSC).

Dataset	Metric	Contrast	Estimate	Lower 95% CI	Upper 95% CI	Adjusted p-value
Original	Shannon diversity	NSC - PMO 0.2 mg/mL	-0.098	-0.749	0.553	0.6539
Rarefied	Shannon diversity	NSC - PMO 0.2 mg/mL	-0.099	-0.746	0.547	0.6473
Original	Shannon diversity	NSC - PMO 2 mg/mL	-0.797	-1.449	-0.146	0.0123
Rarefied	Shannon diversity	NSC - PMO 2 mg/mL	-0.811	-1.458	-0.165	0.0103
Original	Shannon diversity	NSC - PMO 20 mg/mL	-0.446	-1.097	0.206	0.1106
Rarefied	Shannon diversity	NSC - PMO 20 mg/mL	-0.456	-1.103	0.191	0.0980
Original	Pielou´s evenness	NSC - PMO 0.2 mg/mL	-0.013	-0.139	0.114	0.7626
Original	Pielou´s evenness	NSC - PMO 20 mg/mL	-0.096	-0.223	0.030	0.0711
Rarefied	Pielou´s evenness	NSC - PMO 2 mg/mL	-0.129	-0.252	-0.005	0.0385
Original	Pielou´s evenness	NSC - PMO 2 mg/mL	-0.129	-0.256	-0.003	0.0409
Rarefied	Pielou´s evenness	NSC - PMO 0.2 mg/mL	-0.016	-0.140	0.107	0.6965
Rarefied	Pielou´s evenness	NSC - PMO 20 mg/mL	-0.094	-0.218	0.029	0.0701
Original	Observed richness	NSC - PMO 0.2 mg/mL	-5.167	-25.068	14.735	0.6223
Rarefied	Observed richness	NSC - PMO 0.2 mg/mL	-3.167	-20.268	13.934	0.6987
Original	Observed richness	NSC - PMO 2 mg/mL	-29.500	-49.401	-9.599	0.0016
Rarefied	Observed richness	NSC - PMO 2 mg/mL	-31.500	-48.601	-14.399	0.0003
Original	Observed richness	NSC - PMO 20 mg/mL	-0.833	-20.735	19.068	0.9005
Rarefied	Observed richness	NSC - PMO 20 mg/mL	-4.000	-21.101	13.101	0.6987

Supplementary Table S5. Summary of genus-level differential abundance analysis results obtained using MaAsLin2.

Differential abundance analysis was performed on absolute abundance data using MaAsLin2 with dose as a fixed effect and donor as a random effect. Genera present in fewer than 50 % of samples (n = 24) were excluded prior to analysis (prevalence threshold = 0.5). A pseudocount of 1 was added to each value to enable log transformation. P-values were adjusted for multiple testing using the Benjamini-Hochberg procedure across all tested genera. Positive coefficients indicate higher abundances relative to the untreated 48 h control, whereas negative coefficients indicate lower abundances after PMO treatment.

Genus	Treatment	Coefficient	Standard error	Non-zero samples	p-value	q-value
Parabacteroides	20 mg/mL	-9.494944619	1.033462806	15	1.29E-08	1.43E-06
Romboutsia	2 mg/mL	5.500012198	0.607060086	17	1.80E-07	8.23E-06
Parabacteroides	2 mg/mL	-7.852966656	1.033462806	15	2.56E-07	8.23E-06
Romboutsia	20 mg/mL	5.28913014	0.607060086	17	2.96E-07	8.23E-06
Lachnospiraceae UCG 009	20 mg/mL	-4.425268995	0.527211725	13	4.74E-07	1.05E-05
Lachnospiraceae UCG 009	2 mg/mL	-4.258602328	0.527211725	13	7.64E-07	1.41E-05
Bacteroides	20 mg/mL	-7.9743427	1.049537849	24	1.62E-06	2.56E-05
Incertae Sedis	20 mg/mL	3.11331023	0.425941687	14	2.57E-06	3.52E-05
Incertae Sedis	2 mg/mL	3.086365297	0.425941687	14	2.85E-06	3.52E-05
Collinsella	20 mg/mL	5.391572359	0.783620167	16	5.23E-06	5.81E-05
Collinsella	2 mg/mL	5.217615366	0.783620167	16	7.63E-06	7.70E-05
F_Coriobacteriales Incertae Sedis	2 mg/mL	3.541913461	0.623479307	13	4.36E-05	4.03E-04
Bacteroides	2 mg/mL	-5.725449313	1.049537849	24	6.64E-05	5.67E-04
F_Coriobacteriales Incertae Sedis	20 mg/mL	3.329387389	0.623479307	13	8.25E-05	6.54E-04
Eggerthella	20 mg/mL	-5.672217424	1.099617576	17	1.17E-04	8.64E-04
Escherichia-Shigella	20 mg/mL	-9.572922652	1.917242601	21	1.61E-04	1.11E-03
Lachnoclostridium	20 mg/mL	-3.891971559	0.812248265	21	2.38E-04	1.55E-03
Escherichia-Shigella	2 mg/mL	-8.784550536	1.917242601	21	3.60E-04	2.22E-03
Lachnospiraceae NK4A136 group	2 mg/mL	1.836058432	0.425793251	14	6.17E-04	3.43E-03
Subdoligranulum	2 mg/mL	3.472192606	0.805474446	20	6.18E-04	3.43E-03
Lachnospiraceae NK4A136 group	20 mg/mL	1.753954983	0.425793251	14	9.10E-04	4.50E-03
Eubacterium hallii group	2 mg/mL	2.683638748	0.653120209	17	9.29E-04	4.50E-03
Subdoligranulum	20 mg/mL	3.308370027	0.805474446	20	9.32E-04	4.50E-03
Eggerthella	2 mg/mL	-4.467614253	1.099617576	17	1.02E-03	4.72E-03
Blautia	20 mg/mL	2.906509946	0.743942467	23	1.40E-03	6.22E-03
Eubacterium hallii group	20 mg/mL	2.516685772	0.653120209	17	1.56E-03	6.67E-03
Faecalibacterium	2 mg/mL	4.057912288	1.068885061	17	1.76E-03	7.22E-03
Blautia	2 mg/mL	2.766785975	0.743942467	23	2.06E-03	8.15E-03
Faecalibacterium	20 mg/mL	3.844673131	1.068885061	17	2.64E-03	1.01E-02
Lachnoclostridium	2 mg/mL	-2.798711708	0.812248265	21	3.60E-03	1.33E-02
Anaerostipes	20 mg/mL	2.016891946	0.605433204	14	4.56E-03	1.63E-02
Anaerostipes	2 mg/mL	1.925986463	0.605433204	14	6.20E-03	2.15E-02
Flavonifractor	20 mg/mL	-1.851578424	0.608860149	14	8.25E-03	2.78E-02
Gordonibacter	20 mg/mL	-2.276134626	0.830235798	16	1.51E-02	4.94E-02
Flavonifractor	0.2 mg/mL	1.633068329	0.608860149	14	1.71E-02	5.41E-02
F_Lachnospiraceae	20 mg/mL	-2.807563038	1.090967436	21	2.12E-02	6.53E-02
Agathobacter	2 mg/mL	1.774078826	0.694285593	13	2.20E-02	6.59E-02
Alistipes	0.2 mg/mL	-3.658946601	1.452615006	17	2.36E-02	6.75E-02
Bilophila	20 mg/mL	-2.572473758	1.022358778	24	2.37E-02	6.75E-02
F_Ruminococcaceae	2 mg/mL	1.983976062	0.808099761	15	2.68E-02	7.43E-02
Bifidobacterium	20 mg/mL	1.542262879	0.642168907	23	2.97E-02	7.89E-02
F_Lachnospiraceae	2 mg/mL	-2.617854584	1.090967436	21	2.98E-02	7.89E-02
Agathobacter	20 mg/mL	1.644141937	0.694285593	13	3.17E-02	8.19E-02
Clostridium sensu stricto 1	2 mg/mL	2.956351946	1.323357083	14	4.11E-02	1.01E-01
Gordonibacter	2 mg/mL	-1.850927611	0.830235798	16	4.15E-02	1.01E-01

Genus	Treatment	Coefficient	Standard error	Non-zero samples	p-value	q-value
UCG 002	2 mg/mL	1.156976774	0.519837049	18	4.18E-02	1.01E-01
Bifidobacterium	2 mg/mL	1.413431929	0.642168907	23	4.38E-02	1.03E-01
Eisenbergiella	20 mg/mL	-1.705247225	0.803592236	13	5.09E-02	1.18E-01
Alistipes	20 mg/mL	-3.023690563	1.452615006	17	5.49E-02	1.24E-01
Slackia	2 mg/mL	-1.882503421	0.95083072	16	6.64E-02	1.47E-01
Slackia	20 mg/mL	-1.859691981	0.95083072	16	6.94E-02	1.51E-01
UBA1819	20 mg/mL	-1.71515012	0.909054093	13	7.87E-02	1.68E-01
Clostridium sensu stricto 1	20 mg/mL	2.421682238	1.323357083	14	8.72E-02	1.83E-01
Bilophila	2 mg/mL	-1.825365573	1.022358778	24	9.44E-02	1.94E-01
Blautia	0.2 mg/mL	1.2766308	0.743942467	23	1.07E-01	2.13E-01
Roseburia	20 mg/mL	-1.743817161	1.018477786	13	1.07E-01	2.13E-01
Flavonifractor	2 mg/mL	-1.026168553	0.608860149	14	1.13E-01	2.19E-01
Eisenbergiella	2 mg/mL	-1.343104312	0.803592236	13	1.15E-01	2.21E-01
F_Ruminococcaceae	20 mg/mL	1.311768945	0.808099761	15	1.25E-01	2.36E-01
Enterorhabdus	20 mg/mL	1.287629362	0.805203319	15	1.31E-01	2.42E-01
Enterorhabdus	2 mg/mL	1.261958325	0.805203319	15	1.38E-01	2.51E-01
Lachnospiraceae UCG 009	0.2 mg/mL	0.820185337	0.527211725	13	1.41E-01	2.52E-01
Lachnoclostridium	0.2 mg/mL	1.222026163	0.812248265	21	1.53E-01	2.70E-01
Streptococcus	2 mg/mL	1.812425734	1.437107438	13	2.22E-01	3.85E-01
Ruminococcus torques group	0.2 mg/mL	1.259086709	1.002014287	22	2.28E-01	3.90E-01
Dialister	20 mg/mL	1.280352252	1.054688051	14	2.44E-01	4.10E-01
Coprococcus	0.2 mg/mL	1.275159313	1.184686986	23	2.99E-01	4.88E-01
Bilophila	0.2 mg/mL	-1.100091861	1.022358778	24	2.99E-01	4.88E-01
Roseburia	2 mg/mL	1.067313849	1.018477786	13	3.11E-01	5.01E-01
Ruminococcus torques group	2 mg/mL	0.966900708	1.002014287	22	3.50E-01	5.55E-01
Roseburia	0.2 mg/mL	0.967784187	1.018477786	13	3.57E-01	5.58E-01
Streptococcus	0.2 mg/mL	1.336459294	1.437107438	13	3.63E-01	5.58E-01
F_Lachnospiraceae	0.2 mg/mL	1.014915074	1.090967436	21	3.67E-01	5.58E-01
Eggerthella	0.2 mg/mL	-1.006376319	1.099617576	17	3.75E-01	5.62E-01
Streptococcus	20 mg/mL	1.270362636	1.437107438	13	3.87E-01	5.73E-01
Eisenbergiella	0.2 mg/mL	0.705766978	0.803592236	13	3.94E-01	5.75E-01
Dialister	2 mg/mL	0.87536922	1.054688051	14	4.20E-01	6.05E-01
Subdoligranulum	0.2 mg/mL	0.641534129	0.805474446	20	4.38E-01	6.21E-01
Ruminococcus torques group	20 mg/mL	0.790774538	1.002014287	22	4.42E-01	6.21E-01
Alistipes	2 mg/mL	-1.115583485	1.452615006	17	4.54E-01	6.28E-01
Adlercreutzia	2 mg/mL	1.026382194	1.347347778	17	4.58E-01	6.28E-01
UBA1819	0.2 mg/mL	-0.645565744	0.909054093	13	4.89E-01	6.61E-01
Romboutsia	0.2 mg/mL	0.392273016	0.607060086	17	5.28E-01	7.06E-01
Adlercreutzia	20 mg/mL	0.770813114	1.347347778	17	5.76E-01	7.40E-01
Dialister	0.2 mg/mL	-0.594029131	1.054688051	14	5.82E-01	7.40E-01
Parabacteroides	0.2 mg/mL	-0.574147434	1.033462806	15	5.85E-01	7.40E-01
Faecalibacterium	0.2 mg/mL	0.592849062	1.068885061	17	5.87E-01	7.40E-01
F_Ruminococcaceae	0.2 mg/mL	0.443460572	0.808099761	15	5.91E-01	7.40E-01
Gordonibacter	0.2 mg/mL	0.452181854	0.830235798	16	5.94E-01	7.40E-01
Agathobacter	0.2 mg/mL	0.37159897	0.694285593	13	6.00E-01	7.40E-01
Dorea	2 mg/mL	0.505645403	1.095989879	22	6.51E-01	7.93E-01
Collinsella	0.2 mg/mL	0.351708768	0.783620167	16	6.60E-01	7.93E-01
Bifidobacterium	0.2 mg/mL	0.284258082	0.642168907	23	6.64E-01	7.93E-01
Anaerostipes	0.2 mg/mL	0.243247056	0.605433204	14	6.94E-01	8.11E-01
Clostridium sensu stricto 1	0.2 mg/mL	0.53064636	1.323357083	14	6.94E-01	8.11E-01
Slackia	0.2 mg/mL	0.358870514	0.95083072	16	7.11E-01	8.22E-01
Incertae Sedis	0.2 mg/mL	0.147912521	0.425941687	14	7.33E-01	8.39E-01
F_Coriobacteriales Incertae Sedis	0.2 mg/mL	0.205258655	0.623479307	13	7.47E-01	8.46E-01
Escherichia-Shigella	0.2 mg/mL	-0.579302911	1.917242601	21	7.67E-01	8.55E-01
Dorea	20 mg/mL	0.32620367	1.095989879	22	7.70E-01	8.55E-01
UCG 002	20 mg/mL	-0.130590919	0.519837049	18	8.05E-01	8.73E-01
UBA1819	2 mg/mL	-0.225798585	0.909054093	13	8.07E-01	8.73E-01

Genus	Treatment	Coefficient	Standard error	Non-zero samples	p-value	q-value
Eubacterium hallii group	0.2 mg/mL	0.159779072	0.653120209	17	8.10E-01	8.73E-01
Coprococcus	20 mg/mL	0.251686554	1.184686986	23	8.35E-01	8.91E-01
Adlercreutzia	0.2 mg/mL	0.258999084	1.347347778	17	8.50E-01	8.99E-01
Enterorhabdus	0.2 mg/mL	0.128098867	0.805203319	15	8.76E-01	9.17E-01
Dorea	0.2 mg/mL	-0.139734313	1.095989879	22	9.00E-01	9.31E-01
Coprococcus	2 mg/mL	0.14204879	1.184686986	23	9.06E-01	9.31E-01
UCG 002	0.2 mg/mL	-0.038868368	0.519837049	18	9.41E-01	9.59E-01
Bacteroides	0.2 mg/mL	0.061571263	1.049537849	24	9.54E-01	9.63E-01
Lachnospiraceae NK4A136 group	0.2 mg/mL	0.015993442	0.425793251	14	9.71E-01	9.71E-01
